# Comprehensive analysis of the *LHT* gene family in tobacco and functional characterization of *NtLHT22* involvement in amino acids homeostasis

**DOI:** 10.3389/fpls.2022.927844

**Published:** 2022-09-13

**Authors:** Zhaowu Li, Junping Gao, Shuaibin Wang, Xiaodong Xie, Zhangying Wang, Yu Peng, Xiaonian Yang, Wenxuan Pu, Yaofu Wang, Xiaorong Fan

**Affiliations:** ^1^Tobacco Research Institute of Technology Centre, China Tobacco Hunan Industrial Corporation, Changsha, China; ^2^State Key Laboratory of Crop Genetics and Germplasm Enhancement, Ministry of Agriculture Key Laboratory of Plant Nutrition and Fertilization in Lower-Middle Reaches of the Yangtze River, Nanjing Agricultural University, Nanjing, China; ^3^China Tobacco Gene Research Center, Zhengzhou Tobacco Research Institute of China National Tobacco Corporation, Zhengzhou, China; ^4^State Key Laboratory of Hybrid Rice, College of Life Sciences, Wuhan University, Wuhan, China

**Keywords:** *Nicotiana tabacum*, *LHT*, bioinformatics, expression profile, amino acids

## Abstract

Amino acids are vital nitrogen (N) sources for plant growth, development, and yield. The uptake and translocation of amino acids are mediated by amino acid transporters (AATs). The AATs family including lysine-histidine transporters (LHTs), amino acid permeases (AAPs), and proline transporters (ProTs) subfamilies have been identified in various plants. However, little is known about these genes in tobacco. In this study, we identified 23 *LHT* genes, the important members of AATs, in the tobacco genome. The gene structure, phylogenetic tree, transmembrane helices, chromosomal distribution, *cis*-regulatory elements, and expression profiles of *NtLHT* genes were systematically analyzed. Phylogenetic analysis divided the 23 *NtLHT* genes into two conserved subgroups. Expression profiles confirmed that the *NtLHT* genes were differentially expressed in various tissues, indicating their potential roles in tobacco growth and development. *Cis*-elements analysis of promoters and expression patterns after stress treatments suggested that *NtLHT* genes probable participate in abiotic stress responses of tobacco. In addition, Knock out and overexpression of *NtLHT22* changed the amino acids homeostasis in the transgenic plants, the contents of amino acids were significantly decreased in *NtLHT22* overexpression plants than wild-type. The results from this study provide important information for further studies on the molecular functions of the *NtLHT* genes.

## Introduction

Nitrogen (N) is the major limiting factor among mineral nutrients for plant growth and development. In natural soil ecosystem, plant roots absorb both inorganic N and organic N including amino acids and peptides (Paungfoo-Lonhienne et al., 2008; Näsholm et al., 2009). Amino acids are the largest pool of dissolved organic N and can be acquired by plant roots. Soil amino acids are mainly derived from peptides of decaying organisms and decomposition of proteins (Kielland et al., 2007). In the soil solution, asparagine, aspartate, glutamine, glutamate, and glycine are the major forms of amino acids, and the concentrations of total free amino acids in the soil may be up to 150 μM (Senwo and Tabatabai, 1998; Weigelt et al., 2005).

In general, inorganic N is converted and transported in the form of amino acids in plants. Therefore, amino acids play crucial role in plants, including regulating root architecture, flowering time, and seed setting rate (Guo et al., 2021). Amino acid transporters (AATs) play vital roles in amino acid transport and translocation. Nowadays, plant AATs have been identified and are grouped into two superfamilies: amino acid-polyamine-choline transporters (APCs) and amino acid/auxin permeases (AAAPs) (Fischer et al., 1998). The APC superfamily includes L-type amino acid transporter (LAT) families and cationic amino acid transporter (CAT) (Tegeder, 2012; Li F. et al., 2021). The AAAP superfamily includes auxin transporters (AUXs), amino acid permeases (AAPs), γ-aminobutyric acid transporters (GATs), lysine and histidine transporters (LHTs), and proline transporters (ProTs) (Okumoto et al., 2002, 2004; Okumoto and Pilot, 2011).

Many AATs have been well researched in *Arabidopsis*. *AtAAP1* is expressed in embryos, amino acid loaded into embryo *via AtAAP1* is important for storage protein synthesis and seed yield (Sanders et al., 2009). *AtAAP2* is expressed in the phloem, disruption of *AtAAP2* increased amino acids allocation to leaves and resulted in higher seed yields (Zhang et al., 2010). *AtAAP6* regulates phloem amino acids composition, in *aap6* mutant, the concentrations of leucine, lysine, aspartate, and phenylalanine were significantly lower than wild-type plants (Hunt et al., 2010). *AtAAP8* plays a crucial role in amino acids loading into the phloem. In *aap8* mutants, amino acids loading into phloem and partitioning to sink tissues were decreased (Schmidt et al., 2007). *AtLHT1*, the first member identified in LHT family, was critical for amino acids uptake. *AtLHT1* is expressed in various plant organs, including roots, flowers, and young leaves. Disruption of *AtLHT1* decreased shoot biomass and seed yield, while overexpression of *AtLHT1* enhanced amino acids uptake and improved the N use efficiency (Chen and Bush, 1997; Hirner et al., 2006). Pathogen infection activates the expression of *AtLHT1*, further studies suggested that *AtLHT1* is a negative regulator of disease resistance, the *lht1* mutants enhanced disease resistance to a broad spectrum of pathogens (Liu et al., 2010). *AtLHT1* is also involved in the uptake of ethylene precursor 1-aminocyclopropane-1-carboxylic acid (ACC), loss-of-function of *AtLHT1* caused dose-dependent resistance to exogenous ACC (Shin et al., 2015). *AtLHT2* is expressed preferentially in floral organs, transgenic expression of *AtLHT2* could rescue the early senescence phenotypes of the *lht1* mutant (Choi et al., 2019).

In rice, at least 85 putative AATs were identified (Zhao et al., 2012). *OsAAP1* is mainly expressed in young panicles, leaves, andaxillary buds, and OsAAP1 protein is located in both the nuclear and plasma membranes. Overexpression of *OsAAP1* increased tiller numbers and filled grain numbers, while *OsAAP1* RNAi lines showed the opposite phenotype (Ji et al., 2020). *OsAAP3* is highly expressed in lateral root, leaf sheath, leaf, panicle, and culm. Overexpression of *OsAAP3* inhibited bud outgrowth and tillering, while downregulation of *OsAAP3* expression promotes bud elongation and boosts grain yield (Lu et al., 2018). *OsAAP5* is mainly expressed in panicles and leaves, *OsAAP5* could transports anionic, neutral, and cationic amino acids. Loss-of-function of *OsAAP5* could increase tiller number and grain yield (Svennerstam et al., 2008; Wang J. et al., 2019). *OsLHT1* is expressed in root hairs and lateral roots and is a key transporter for root amino acids uptake (Guo et al., 2020a). Loss-of-function of *OsLHT1* decreased amino acids allocation from root to shoot, thereby markedly reduced shoot growth and grain yields (Wang X. et al., 2019). The *japonica* subspecies take up aspartate 1.5-fold more efficiently than *indica* subspecies. Moreover, the expression levels of *OsLHT1* in *japonica* is higher than that in *indica*. It has been shown that the expression of *OsLHT1* and root aspartate uptake was positive correlation (Guo et al., 2020b).

Tobacco (*Nicotiana tabacum* L.) is one of the most important economic crops, it is cultivated worldwide and used as a model for plant biology research. AATs functions in long distance amino acids transport and are essential for plant growth and development. Lots of *AAT* genes, such as *AAP, ProT*, and *LHT* genes have been well studied and characterized in *Arabidopsis*, rice, tea, and other plants. However, the studies of AATs is limited in tobacco. In this study, we identified *LHT* gene family, the important members of AATs, in the tobacco genome with bioinformatics methods. The phylogenic relationships, gene structures, chromosomal distributions, syntenic regions, and *cis*-regulatory elements in these *NtLHT* genes were analyzed. Moreover, the expression profiles of *NtLHT* genes in different tissues and in response to drought and cold stresses were investigated. Our findings provide useful information for further functional research of *NtLHT* genes in tobacco.

## Materials and methods

### Plant materials and growth conditions

The *N. tabacum* L. K326 plants were cultured in a greenhouse with conditions described in previous report (Li Z. et al., 2021). For expression profiles of the *NtLHT* genes in different tissues, total RNA was isolated from the roots, axillary buds, stems, flowers, and leaves of tobacco at the flowering stage. For drought treatment, 3-week-old seedlings were not watered, the obviously wilted plants were termed as drought stress. For cold treatment, 3-week-old seedlings were exposed to 4°C for 5 h (Xie et al., 2021).

### Annotation and identification of putative *NtLHT* genes

For *NtLHT* identification, the protein sequences of AtLHTs were retrieved from TAIR^1^ (Rhee et al., 2003), the obtained sequences were then used as a query to search putative *NtLHT* genes from the China tobacco genome database (data not shown). Top hits for putative NtLHT proteins were retained on the basis of high scores (score ≥ 0) and low *E*-values (*E* value ≤ 0.1), the NCBI conserved domain database (CDD)^2^ was used to analyze the conserved domain (PF01490) of candidate NtLHT proteins (Rabby et al., 2022). After manually removing the redundant sequences, 23 *NtLHT* genes were identified and used for further bioinformatics and expression analysis.

### Physicochemical characterization, transmembrane, and protein structure analysis

The physiochemical properties of NtLHT proteins were predicted using the ExPASy tool (Artimo et al., 2012). The subcellular location of NtLHT proteins were predicted using The WOLF PSORT II program.^3^ The PROTTER 1.0 was used to predict the presence of transmembrane helices,^4^ and the tertiary protein structures of NtLHT proteins were analyzed by PHYRE server v2.0.^5^

### Phylogeny analysis, gene structure, and motifs analysis

The protein sequences of LHT from tobacco (NtLHT), tea (CsLHT), *Arabidopsis* (AtLHT), and rice (OsLHT) were used to construct a phylogenetic tree in MEGA X program *via* the neighbor-joining (NJ) method with 1,000 bootstrap replicates (Supplementary Table 1). The coding sequence of each NtLHT (Supplementary Table 2) was aligned with its corresponding genomic sequence to identify the exon–intron structure by GSDS tool.^6^ The conserved motifs of NtLHT proteins were analyzed using MEME tool with default parameter (Bailey et al., 2006).

### Chromosomal mapping and gene duplication analysis

The chromosomal positions of *NtLHT* genes were mapped using MG2C 2.0.^7^ Segmental and tandem duplicated gene pairs among the tobacco, tea, *Arabidopsis*, and rice genomes were conducted using MCScanX. The collinearity map was drawn with Circos. The non-synonymous substitution (Ka) and synonymous substitution rate (Ks) of duplicated genes were determined using KaKs Calculator 2.0.

### *Cis*-regulatory elements analysis

To assess the *cis*-regulatory elements of the *NtLHT* promoters, the 2 kb DNA sequence upstream from the transcription start site (start codon ATG) of *NtLHT* genes were extracted, the obtained sequences were analyzed using the PlantCARE program^8^ and classified according to their regulatory functions.

### Expression analysis of *NtLHT* genes

Total RNA was isolated from plant tissue samples using the Plant RNA Kit (Transgen, Beijing, China). The first strand cDNA synthesis was performed according to the manufacturer’s directions (Thermo Fisher Scientific, Waltham, MA, United States). The quantitative real time PCR (qRT-PCR) was carried out using the CFX96 (Bio-Rad, Hercules, CA, United States) with 20 μL volume (containing 1 μL of 1:10 diluted cDNA, 200 nM of each gene-specific primer, and SYBR Green Mix from Bio-Rad). PCR cycling parameters were set as following: 95°C for 5 min, 40 cycles of 10 s at 95°C and 30 s at 60°C, then a final melting curve at 65°C for 5 s. The stably expressed gene *NtL25* (ribosomal protein gene) was used as the internal control for data normalization. Expression data was carried out with three biological and technical replicates and calculated using the 2^–ΔΔCt^ method (Schmittgen and Livak, 2008). The primers used in this study are listed in Supplementary Table 3.

### Subcellular localization analysis

The coding sequence (CDS) of *NtLHT22* without the stop codon was amplified and cloned into pEarlyGate101 vector (Invitrogen, Carlsbad, CA, United States) to produce *35S*: *NtLHT22-YFP* construct, the empty pEarlyGate101 vector was used as a control. Tobacco protoplasts preparation and transformation for subcellular localization experiments were carried out as previously described (Schweiger and Schwenkert, 2014). After transformation, the fluorescence signals was observed *via* Leica SP8-X confocal laser scanning microscope (Leica, Wetzlar, Germany).

### Plasmid construction and tobacco transformation

*NtLHT22* was amplified and cloned into the modified overexpression vector pEarlyGate101 (Invitrogen, Carlsbad, CA, United States) to produce *35S*: *NtLHT22-Myc* construct. The CRISPR/Cas9 vector was constructed as previously described (Gao et al., 2018). The CRISPR-P 2.0 software^9^ was used to design the target sites of *NtLHT22*. Two 20 bp DNA target sites were synthesized and inserted to the pORE-Cas9 binary vector, the resulting constructs was verified *via* PCR and sequencing. The overexpression and pORE-Cas9 vector was transformed into *Agrobacterium tumefaciens* (LBA4404), respectively. The *Agrobacterium*-mediated transformation and regeneration of transgenic tobacco plants were carried out as previously described (Wang et al., 2018; Supplementary Figure 1). Two independent knockout and overexpression lines of *NtLHT22* were identified and used for subsequent experimental studies.

### Free amino acid analysis

Free amino acid contents were measured according to the ninhydrin method with some modifications (Fang et al., 2013). 2.0 g sample was placed in 80% ethanol (10.0 mL) at 95°C for 20 min. The collected extracts were placed at 80°C in a drying oven to remove the ethanol, the residues were re-dissolved in 1 ml water. After centrifugation at 12000 *g* for 15 min, the supernatant was filtered through a 0.45 μm membrane. Amino acid contents were determined using an LA8080 automatic amino acid analyzer (Hitachi, Tokyo, Japan).

## Results

### Genome-wide identification and protein properties of *NtLHT*

To investigate the *LHT* genes in tobacco, we employed 10 AtLHT protein sequences as queries for BLAST search. As a result, 23 *LHT* genes were obtained from the tobacco genome. These genes were named in the order of their locations on the chromosomes and scaffolds. The gene and CDS lengths of NtLHTs ranged from 912 to 17381 bp and 912 to 1716 bp, respectively (Table 1). The length of NtLHT proteins ranged from 303 to 571 amino acids with a molecular weight of 33.4–61.9 kDa, and their pIs ranged from 5.74 to 9.51. The predominant amino acid residues were valine, alanine, and leucine. The instability index for most of the proteins (77.8%) were less than 40. Apart from these, the GRAVY values of all NtLHT proteins were positive, indicating that all NtLHT proteins were hydrophobic. The predicted subcellular locations suggested that most of the NtLHT proteins were localized in the plasma membrane, cytoplasm, and vacuoles (Table 2).

**TABLE 1:** Detailed information of *NtLHT* gene families.

Genes	Gene ID	Chromosome no.	Start site	End site	Gene length (bp)	CDS (bp)	ORF (aa)
*NtLHT1*	Ntab0805870	3	2263184	2267208	4024	1317	438
*NtLHT2*	Ntab0130350	6	88937717	88940844	3127	1716	571
*NtLHT3*	Ntab0598290	6	146913529	146916409	2880	1107	368
*NtLHT4*	Ntab0341790	8	94412713	94415394	2636	1329	442
*NtLHT5*	Ntab0041820	17	36383061	36385553	2492	1287	428
*NtLHT6*	Ntab0164910	17	63016659	63019299	2640	1200	399
*NtLHT7*	Ntab0164970	17	61467595	61471175	3580	1368	455
*NtLHT8*	Ntab0925250	18	32684675	32687761	3086	1536	511
*NtLHT9*	Ntab0131130	19	42043755	42049667	5912	1488	495
*NtLHT10*	Ntab0565950	20	61338513	61355894	17381	1617	538
*NtLHT11*	Ntab0158190	21	71880185	71882999	2814	1329	442
*NtLHT12*	Ntab0028150	Ntab_scaffold_1065	527772	531589	3817	1317	438
*NtLHT13*	Ntab0299590	Ntab_scaffold_1896	118582	122318	3736	1320	439
*NtLHT14*	Ntab0308410	Ntab_scaffold_193	695819	699686	3867	1332	443
*NtLHT15*	Ntab0402760	Ntab_scaffold_2334	54252	57234	2982	1509	502
*NtLHT16*	Ntab0449550	Ntab_scaffold_2560	419100	423978	4878	1575	524
*NtLHT17*	Ntab0543030	Ntab_scaffold_3056	259747	264911	5164	1368	455
*NtLHT18*	Ntab0587850	Ntab_scaffold_3350	263976	268854	4878	1575	524
*NtLHT19*	Ntab0678610	Ntab_scaffold_423	1394974	1399957	4983	1575	524
*NtLHT20*	Ntab0739090	Ntab_scaffold_524	762837	763749	912	912	303
*NtLHT21*	Ntab0814760	Ntab_scaffold_656	520549	524400	3851	1332	443
*NtLHT22*	Ntab0818090	Ntab_scaffold_661	743641	749851	6210	1368	455
*NtLHT23*	Ntab0931880	Ntab_scaffold_88	2449996	2452073	2077	1107	368

**TABLE 2:** Amino acid composition and physiochemical characteristics of NtLHT proteins.

Proteins	MW	pI	Major amino acid %	Instability index	GRAVY	Localization predicted
NtLHT1	48.8	8.92	V(10.0), A(8.7), G(8.0)	38.52	0.445	plas, vacu
NtLHT2	61.9	9.37	L(11.9), A(10.2), S(8.8)	32.76	0.482	plas, vacu
NtLHT3	41.1	9.43	V(10.1), L(8.7), S(7.9)	37.75	0.270	plas, vacu, ER
NtLHT4	49.7	8.34	V(10.2), L(8.6), I(8.1)	32.29	0.482	plas, ER
NtLHT5	47.8	9.51	V(10.0), S(9.6), L(8.6)	34.37	0.409	plas, ER, vacu
NtLHT6	60.2	7.60	V(11.3), I(8.7), L(8.4)	27.21	0.567	plas, cyto, vacu
NtLHT7	51.0	8.75	V(11.2), A(7.7), G(7.5)	35.26	0.460	plas, ER, vacu
NtLHT8	56.5	9.05	L(12.7), I(9.0), S(8.8)	34.66	0.518	plas, cyto, ER
NtLHT9	54.7	8.88	L(10.5), S(9.3), G(8.1)	41.13	0.464	plas, vacu, ER
NtLHT10	44.8	8.37	V(10.5), L(9.5), I(9.3)	26.53	0.621	plas, cyto, vacu
NtLHT11	49.6	8.07	V(10.0), L(8.4), I(8.1)	35.16	0.502	plas, ER
NtLHT12	48.8	8.92	V(10.0), A(8.4), G(8.0)	38.15	0.451	plas, vacu
NtLHT13	49.2	8.82	L(9.1), I(8.9), V(8.7)	39.49	0.444	plas, ER, gol
NtLHT14	49.2	9.37	V(9.0), L(8.8), G(8.6)	39.93	0.456	plas, ER
NtLHT15	55.4	9.07	L(12.5), S(9.4), I(9.4)	35.10	0.529	plas, cyto
NtLHT16	57.8	9.41	L(11.8), S(9.0), A(8.6)	35.10	0.457	plas, cyto, chlo
NtLHT17	41.1	9.43	V(10.1), L(8.7), S(7.9)	37.75	0.270	plas, vacu, ER
NtLHT18	57.8	9.41	L(11.8), S(9.0), A(8.6)	35.10	0.457	plas, cyto, chlo
NtLHT19	57.8	9.41	L(11.8), S(9.0), A(8.6)	35.10	0.457	plas, cyto, chlo
NtLHT20	33.4	5.74	V(10.9), G(8.6), A(7.9)	29.25	0.312	plas, cyto
NtLHT21	49.3	9.41	V(9.3), L(9.0), G(8.4)	41.32	0.487	plas, ER
NtLHT22	51.0	9.25	V(9.7), I(9.0), A(8.4)	34.01	0.515	plas, vacu, ER
NtLHT23	41.0	9.48	V(10.1), S(8.4), A(8.4)	32.98	0.316	plas, ER, vacu

MW, molecular weight (kDa); pI, isoelectric point; GRAVY, grand average of hydropathicity; V, Val; I, Ile; A, Ala; G, Gly; S, Ser; L, Leu; Plas, plasma membrane; Vacu, vacuoles; ER, endoplasmic reticulum; Cyto, cytoplasm; Gol, golgi; Chlo, chloroplast.

To comprehensive understand the information of NtLHT proteins, the transmembrane regions were predicted by PROTTER 1.0. The number of transmembrane regions in NtLHT proteins ranged from 5 to 11 (Supplementary Figure 2). The predicted protein structures for all NtLHT proteins revealed the presence of α helices, random coils, extended strands, and β turns. All NtLHT proteins have α helices, while β turns were the least common (Supplementary Figure 3).

### Phylogenetic analysis of lysine-histidine transporter family in tobacco, tea, *Arabidopsis*, and rice

**FIGURE 1:**
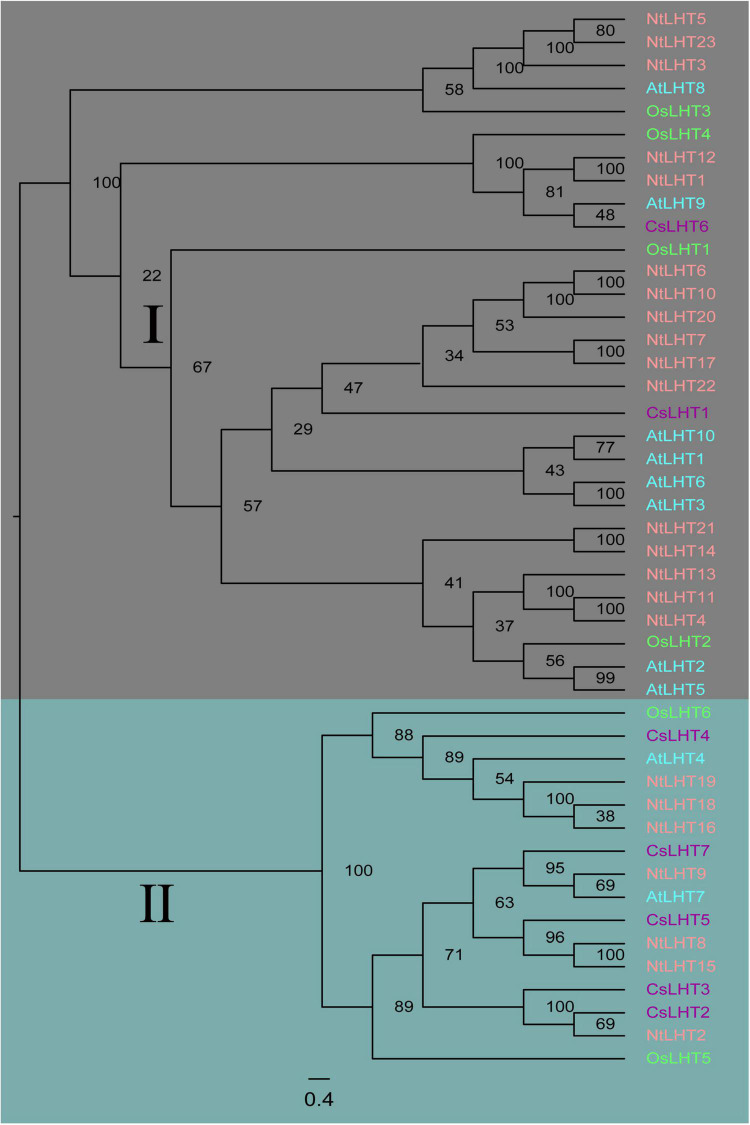
Phylogenetic relationship of LHT proteins from tobacco, tea, *Arabidopsis*, and rice. The LHT proteins were divided into two subgroups (marked as I and II), and distinguished by different colors: NtLHT labeled in pink, CsLHT labeled in purple, AtLHT labeled in cyan, and OsLHT labeled in green.

To explore the evolutionary relationships among *LHT* genes from Tobacco, tea, *Arabidopsis*, and rice, an unrooted NJ tree was constructed based on alignment of 23 *NtLHT* genes in tobacco, 7 *CsLHT* genes in tea, 10 *AtLHT* genes in *Arabidopsis*, 6 *OsLHT* genes in rice. According to the created phylogenetic tree, these *LHT* genes could be classified to two subgroups. Subgroup I contained *AtLHT1/2/3/5/6/8/9/10, OsLHT1/2/3/4, CsLHT1/6*, and *NtLHT1/3/4/5/6/7/10/11/12/13/14/17/20/21/22/23*. Subgroup II contained *AtLHT4/7, OsLHT5/6, CsLHT2/3/4/5/7*, and *NtLHT2/8/9/15/16/18/19* (Figure 1). Therefore, subgroup I had the largest number of *LHT* members in *Arabidopsis* (8 genes), rice (4 genes), and tobacco (16 genes), whereas subgroup II had more *LHT* members from tea (5 genes).

### Gene structure and conserved motif analysis

**FIGURE 2:**
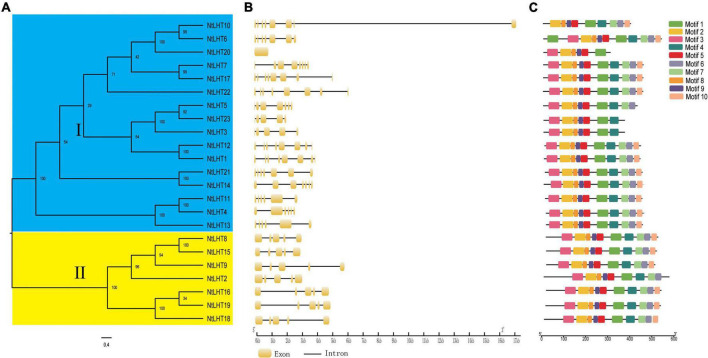
Phylogenetic relationship, exon–intron structures, and conserved motifs of *NtLHT* gene. **(A)** The phylogenetic tree was constructed based on NtLHT protein sequences, different subgroups were highlighted by different colors (subgroup I in gray, subgroup II in blue). **(B)** Exon–intron distribution of *NtLHT* genes. **(C)** Conserved motifs analysis. Different colors represent different motifs.

To clarify the evolutionary relationships of *NtLHT* genes, a phylogenetic tree was generated between *NtLHT* genes. In concordance with the previous results, all the *NtLHT* genes were divided into two subgroups. Subgroup I contained 16 *NtLHT* genes, and subgroup II contained 7 *NtLHT* genes (Figure 2A). The gene structure analysis of *NtLHT* was performed by the GSDS program. The number of exons in subgroup I ranged from 0 to 8, while each member of subgroup II contained 5 exons (Figure 2B). The conserved motif analysis of NtLHT proteins was captured by MEME software. Several motifs were widespread among NtLHT proteins, such as motif 1, 2, 3, 4, 8, 9. The number of conserved motifs in subgroup I ranged from 6 to 11, while most of the members in subgroup II, except NtLHT2, contained 10 motifs (Figure 2C). These results indicating that subgroup II members is more conserved than subgroup I.

### Chromosomal locations and evolutionary analysis of *NtLHT* genes

**FIGURE 3:**
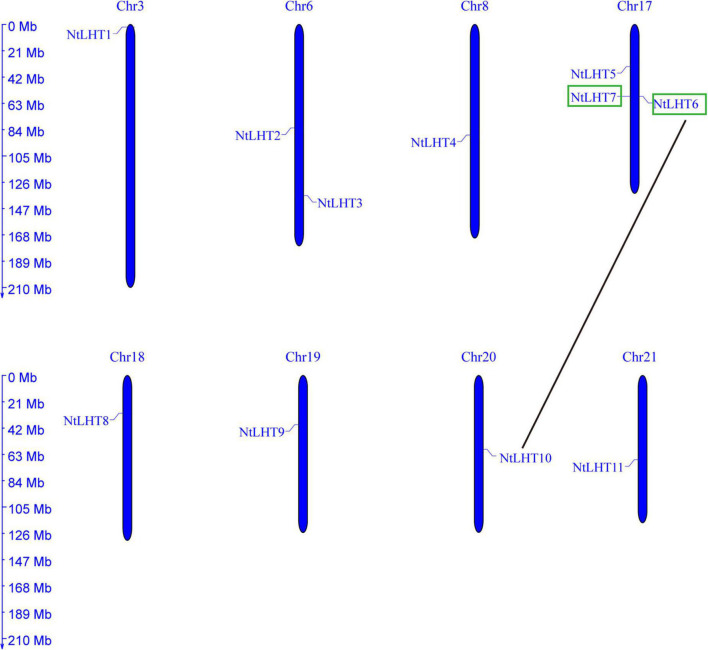
Chromosomal locations and duplication of *NtLHT* genes. Segmental duplication of *NtLHT* genes are connected by black line and green box shows tandem duplicated gene pairs.

The chromosomal distribution of *NtLHT* genes in the tobacco genome were further determined. In the tobacco genome, 11 *NtLHT* genes were unevenly distributed in 8 chromosomes (Chr). There were three *NtLHT* genes on Chr 17, two *NtLHT* genes on Chr 6, Chr 3, 8, 18, 19, 20, and 21 contained one *NtLHT* gene each. The remaining 12 *NtLHT* genes could not be mapped on chromosomes but mapped on some scaffolds (Figure 3).

**FIGURE 4:**
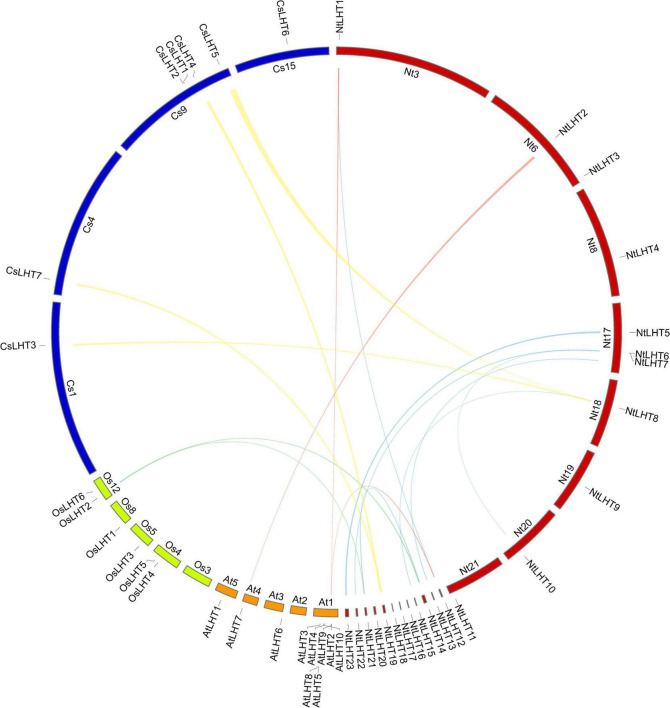
Collinear analysis of *LHT* genes between tobacco, tea, *Arabidopsis*, and rice. Blue lines highlight the segmental duplicated *NtLHT* genes. Yellow, cyan, and red lines highlight the collinear gene pairs between tobacco and tea, tobacco and rice, tobacco and *Arabidopsis*, respectively.

Tandem and segmental duplicates play significant roles for the evolution of species (Xu et al., 2012). Two genes (*NtLHT6* and *NtLHT7*) were tandemly duplicated, while six pairs (*NtLHT1/NtLH12, NtLHT5/NtLHT23, NtLHT6/NtLHT10, NtLHT6/NtLHT22, NtLHT8/NtLHT15*, and *NtLHT14/NtLHT21*) were segmental duplicated (Figure 4). The Ka/Ks values of all tandem and segmental duplicated gene pairs were less than 1 (Supplementary Table 4), indicating that these *NtLHT* genes were evolved under the influence of purifying selection.

We constructed a collinearity plot to investigate the orthologous *LHT* genes in tobacco, tea, *Arabidopsis*, and rice (Figure 4). Two collinear gene pairs were identified between tobacco and rice, the collinear relationships between them were many-to-one matches. Three collinear gene pairs were identified between tobacco and *Arabidopsis*, the collinear relationships between them include many-to-one matches and one-to-one matches. Four collinear gene pairs were identified between tobacco and tea, the collinear relationships between them were one-to-many matches (Supplementary Table 5).

### Tissue-specific expression profiles of *NtLHT* genes

**FIGURE 5:**
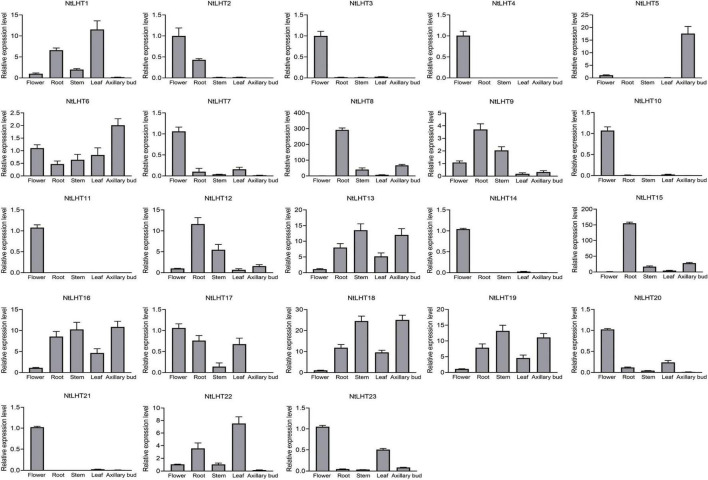
Analysis of *NtLHT* expression levels in various tissues. Expression levels in flowers were set to one. The data were presented as means ± SDs (*n* = 3).

The gene expression profiling in different tissues could provide preliminary clues for their function. To explore the tissue specific expression profiles of *NtLHT* genes, different tissues from tobacco were analyzed, including root, stem, leaf, flower, and axillary bud. The *NtLHT* genes showed diverse expression patterns among different tissues. *NtLHT6/13/16/18/19* were expressed in all these tissues. Ten *NtLHT* genes, *NtLHT2/3/4/7/10/11/14/20/21/23* showed high expression in flower, among which *NtLHT2* also displayed high expression in root and *NtLHT23* had high expression in leaf. *NtLHT8/9/12/15* had the highest expression levels in root. *NtLHT1* and *NtLHT22* showed high expression in leaf. Notably, *NtLHT5* was highly expressed in axillary bud (Figure 5). The results showed that the expression of *NtLHT* have a certain preference and most of these genes were highly expressed in flower, root, and leaf.

### *Cis*-regulatory elements analysis of *NtLHT* genes

**FIGURE 6:**
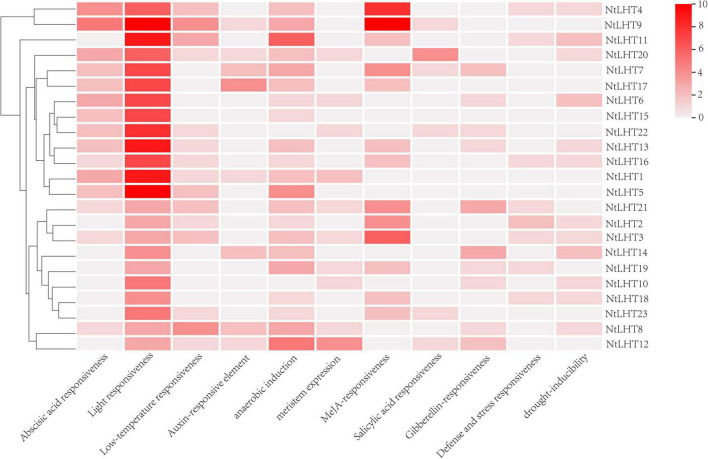
*Cis*-element analysis in the *NtLHT* genes promoter. The color represent the numbers of *cis*-element in *NtLHT* genes promoter.

To explore the regulatory pathways of *NtLHT* genes, we isolated the 2 kb upstream sequences of the *NtLHT* genes for *cis*-element analysis. The largest number of *cis*-elements identified in the *NtLHT* genes promoter was involved in light-responsiveness. Moreover, *cis*-elements associated with anaerobic induction were detected in the promoters of most of the *NtLHT* genes. Apart from these, *cis*-elements associated with hormone response (e.g., abscisic acid, auxin, MeJA, salicylic acid, and gibberellins), stress response (e.g., low temperature, defense and stress, and drought), and meristem expression were also detected in the promoter sequences of *NtLHT* genes (Figure 6). The diversity of *cis*-elements suggest that *NtLHT* genes performed multiple functions in physiological and biological processes.

### Expression profiling of *NtLHT* genes under cold and drought stress

**FIGURE 7:**
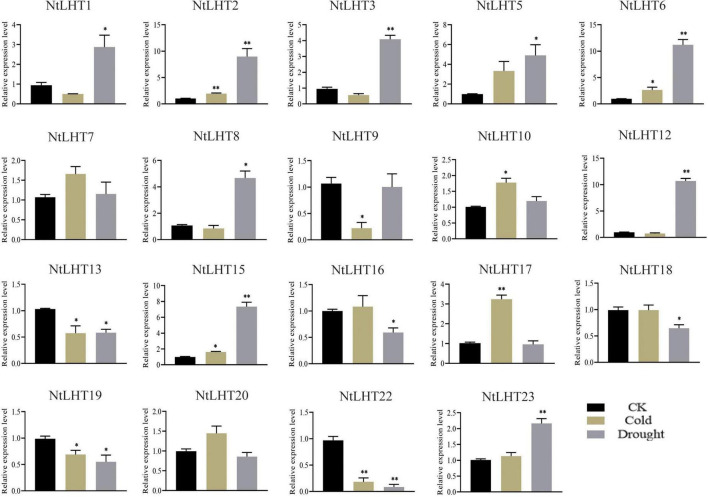
The expression profiles of *NtLHT* genes under cold and drought treatments. Seedlings grown under normal conditions used as controls. The data were presented as means ± SDs (*n* = 3). Significant differences was detected using Student’s *t*-test. *P < 0.05, ^**^P < 0.01.

To analyze the roles of *NtLHT* genes to cold and drought stress, we analyze the expression profiling of *NtLHT* genes after cold and drought treatment. The expression levels of most *NtLHT* genes were differently changed under cold or drought stress (Figure 7). Under cold stress, the expression of *NtLHT2/6/10/15/17* were significantly upregulated, the expression of *NtLHT9/13/19/22* were significantly downregulated. After drought treatment, the expression of *NtLHT1/2/3/5/6/8/12/15/23* were significantly upregulated, while the expression of *NtLHT13/16/18/19/22* were significantly downregulated. In addition, the expression of *NtLHT7* and *NtLHT21* were not significantly changed after cold and drought treatment. Furthermore, four genes (*NtLHT4/11/14/21*) highly expressed in flower were not detected under drought and cold stresses. The results implied that *NtLHT* genes may play distinctive roles in plant response to environmental abiotic stresses.

### Subcellular localization analysis of *NtLHT22*

**FIGURE 8:**
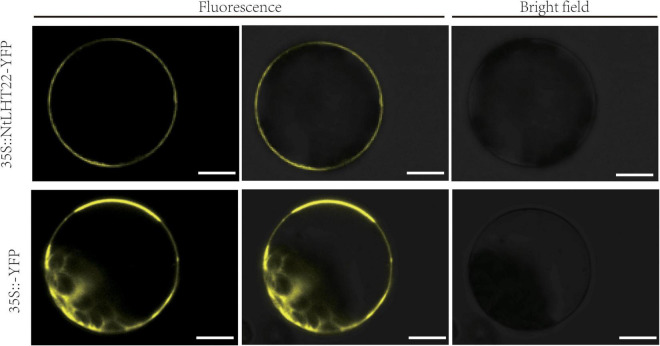
Subcellular localization of NtLHT22 proteins. The coding sequence (CDS) of NtLHT22 was inserted into pEarlyGate101 vector and transiently expressed in tobacco protoplasts. Bar = 10 μm.

To explore the potential functions of the *NtLHT* genes, the subgroup I member *NtLHT22* was selected for subcellular localization analysis. The *35S: NtLHT22-YFP* vector and the *35S*:*YFP* control vector were transiently expressed in tobacco protoplasts, subsequently, the subcellular localization of the YFP signal was detected by confocal laser scanning microscope. In control, the YFP signal was spread throughout the whole protoplasts. In contrast, the signal of the YFP protein fused to NtLHT22 was only observed in the plasma membrane, suggesting that NtLHT22 was targeted to the plasma membrane (Figure 8).

### *NtLHT22* is involved in amino acids homeostasis in tobacco

**FIGURE 9:**
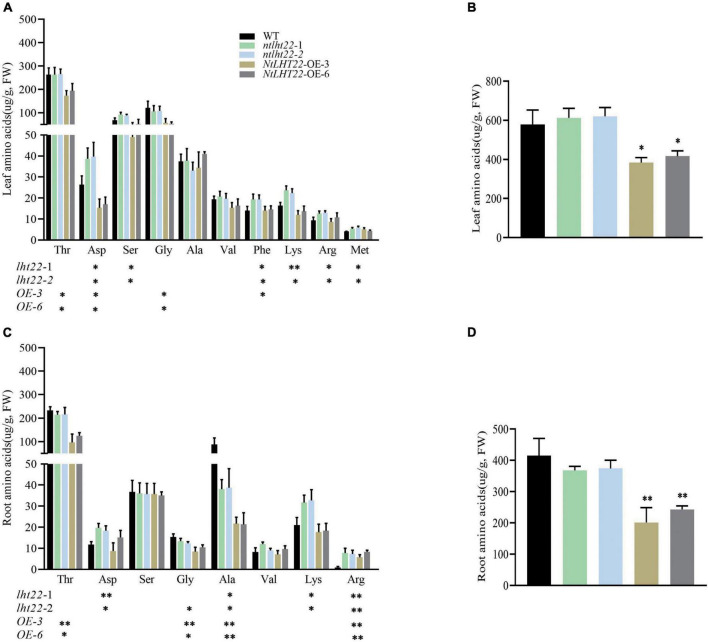
Analysis of amino acids levels of *NtLHT22* overexpression, knock out, and wild-type plants. **(A,B)** Individual and total amino acids content in leaf. **(C,D)** Individual and total amino acids content in roots. FW, fresh weight. The data were presented as means ± SDs (*n* = 3). Significant differences was detected using Student’s *t*-test. *P < 0.05, ^**^P < 0.01.

To further explore the functions of *NtLHT22* in amino acids homeostasis, transgenic plants with overexpressing and knock out of *NtLHT22* were generated, respectively. Two overexpression (OE3 and OE6) and two mutant lines (*ntlht22*-1 and *ntlht22*-2)were chosen for further experiments (Supplementary Figures 4, 5). In leaf tissues, threonine, glycine, serine, aspartic acid, and alanine were the main amino acids. Compared with WT, the levels of six individual amino acids were significantly increased in *ntlht22* mutants, including aspartic acid, serine, phenylalanine, lysine, arginine, and methionine, while the contents of threonine, aspartic acid and glycine were decreased in *NtLHT22-*OE plants (Figure 9A). In root tissues, the levels of threonine, alanine and serine were decreased in *NtLHT22-*OE plants than WT. In *ntlht22* mutants roots, the levels of alanine was decreased but the levels of aspartic acid and lysine were increased than WT. In addition, the content of arginine in *NtLHT22-*OE plants and *ntlht22* mutants roots were significantly higher than WT (Figure 9B). The amino acids content were significantly reduced in *NtLHT22-*OE plants than WT, however, total of amino acids were not changed between *ntlht22* mutants and WT (Figures 9C,D). Taken together, the amino acids profile was significantly changed in *NtLHT22-*OE plants and *ntlht22* mutants, suggesting that *NtLHT22* is participate in amino acids homeostasis in tobacco.

## Discussion

Amino acids are important carriers of N exchange in plants, the absorption and translocation of amino acids are mainly mediated by AATs (Ortiz-Lopez et al., 2000). AATs play significant roles in plant growth and development, including long distance transport and translocate of amino acids, response to biotic and abiotic stresses (Tegeder and Ward, 2012; Ma et al., 2016). The functions of AATs in various plants have been characterized in detail. In potato, *StAAP1* is induced during leaf maturation and the amino acid contents are decreased with reduced *StAAP1* expression (Koch et al., 2003). In *Vicia faba, VfAAP1* is highly expressed in the cotyledons and shows moderate expression in other sink tissues (Miranda et al., 2001). In *Arabidopsis, AtProT* is highly expressed in pollen and the epidermides of leaves, *AtProT1* and *AtProT2* were shown to facilitate the uptake of proline (Lehmann et al., 2011). In rice, *OsProT1* is mainly expressed in roots, stems, and flowers and specifically mediated the transport of L-proline (Igarashi et al., 2000). OsLHT1 participate in root uptake and root-to-shoot allocation of acidic and neutral amino acids, loss-of-function of *OsLHT1* significantly inhibited plants development and reduced seed yields (Wang X. et al., 2019). *LHT* gene family is an important part of *AAT* gene family, however, *LHT* gene family had not been well characterized in tobacco. In this study, we performed a comprehensive identification, classification, and expression profile to study *LHT* gene family in tobacco.

Here, 23 *NtLHT* genes were identified from the tobacco genome. Physicochemical analysis showed that NtLHT proteins exhibited similar physical properties. For example, all NtLHT proteins were alkaline (pI > 7) and hydrophobic (Table 2). The 3D protein structure analysis indicated that NtLHT proteins have highly conservative structure characterized by several α helices, random coils, extended strands, and β turns (Supplementary Figure 3). *NtLHT* genes were divided into two subgroups (subgroup I and subgroup II) and subgroup I contained more *LHT* genes than subgroup II (Figure 1).

Gene structure is closely related with gene evolution and can provide useful information for the study of gene function (Guo et al., 2013). The number of introns in subgroup I ranged from 0 to 8, in subgroup II, all members of *NtLHT* genes contained 5 exons, indicating that the gene structure in subgroup I is more conserved than subgroup II (Figure 2B). We identified ten conserved motifs in NtLHT proteins. The number of conserved motifs in subgroup I ranged from 6 to 11, whereas all NtLHT proteins in subgroup II, except NtLHT2, contained 10 motifs (Figure 2C). Together, these results show that the number of exons/introns and motifs in *NtLHT* gene family were closely related with function conservation or diversification.

Gene duplication, such as whole genome duplication, tandem duplication, and segmental duplication, is the most important pathway for the expansion and evolution of gene families. Moreover, segmental duplications were more important than tandem duplications for the expansion of gene family (Vision Todd et al., 2000; Paterson et al., 2010; Zhang et al., 2020). We obtained one pair of tandem duplicated *NtLHT* genes and six pairs of segmental duplicated *NtLHT* genes in tobacco genome (Figure 4), these results corroborates the previous studies. Additionally, the Ka/Ks values of these duplicated gene pairs were less than 1 (Supplementary Table 4), indicating that duplicated genes have evolved under the influence of purifying selection.

To better understand the functions of *NtLHT* genes, we analyzed the expression profiles of these genes in different tissues (Figure 5). Several *NtLHT* genes (i.e., *NtLHT6* and *NtLHT16*) were highly expressed in most tissues analyzed. Some *NtLHT* genes were highly expressed in specific tissues (i.e., *NtLHT5* in axillary bud, *NtLHT3/4/10*/*11/14* in flower, *NtLHT8* and *NtLHT15* in root), suggesting that these genes may play different physiological functions in different tissues. *Cis*-element contribute to the regulation of gene expression, therefore, the analysis of *cis*-elements is helpful for gene functional characterization. Light-responsiveness elements were ubiquitously identified in the promoter region of *NtLHT* genes, indicating that light might regulate the expression of *NtLHT* genes. In addition, *cis*-elements related to hormone response and stress response were identified in most of the *NtLHT* genes (Figure 6), suggesting that *NtLHT* genes play essential roles in plant growth and development. According to *cis*-elements analysis of *NtLHT* genes, we explored the expression profiling of these genes under cold and drought treatment. The results indicated that 9 and 14 *NtLHT* genes responded to cold and drought stress, respectively. Notably, the expression levels of *NtLHT4*/*11*/*14*/*21* were not detected after cold and drought treatment (Figure 7). These results indicate that the expression of *NtLHT* genes might be regulated by several *cis*-elements, and there also exist unidentified *cis*-elements to regulating the expression of these genes under cold and drought stresses.

Various membrane-localized transporters involved in amino acids uptake, accumulation and translocation, in addition, efficient use of the N inside the crops for quality and yield relies on the proper amino acid metabolism. Previous work suggests that amino acid distribution processes in leaves affect N uptake, probably *via* changes in amino acid metabolism and shoot-to-root signaling processes (Tegeder, 2014). Genetic manipulation of AATs is potentially able to alter plant performance with coordinating amino acid assimilation and metabolism, and ultimately influence quality and yield. A large amount of N is needed in the process of tobacco growing, and the content of amino acids in leaves is also closely related to the quality of tobacco. Our results indicated that overexpression and mutant of *NtLHT22* changed the amino acids profile in leaf and root, and the accumulation of amino acids were significantly decreased in *NtLHT22-*OE plants than WT (Figures 9A–D). Therefore, *NtLHT22* might be involved in the homeostasis of amino acids in different tissues, the alterations of amino acids accumulaion may trigger the changes in N metabolism and quality. Furthermore, *NtLHT22* could be used as a potential gene for quality and yield breeding, and future studies will explore the essential roles of *NtLHT22* in quality as well as N use efficiency.

## Conclusion

In summary, we discovered 23 *NtLHT* genes and analyzed their physicochemical characteristics, phylogenetic relationships, chromosomal locations, and *cis*-elements. We also explored the expression profiles of *NtLHT* genes in different tissues and under abiotic stresses. Furthermore, overexpression and mutant of *NtLHT22* in tobacco changed the amino acids profile, suggesting that *NtLHT22* might be involved in amino acids homeostasis. Overall, our studies provided useful information for the role of *NtLHT* and laid a solid foundation for further investigations of the biological mechanisms of *NtLHT* genes.

## Data availability statement

The original contributions presented in this study are included in the article/Supplementary material, further inquiries can be directed to the corresponding authors.

## Author contributions

ZL and JG conceived and designed the study. SW, XX, and ZW conducted the bioinformatics analysis. YP, XY, WP, and YW assisted in data collection. ZL and XF wrote the manuscript. All authors read and approved the manuscript.

## Footnotes

1
http://www.arabidopsis.org

2
https://www.ncbi.nlm.nih.gov/cdd/

3
http://www.genscript.com/wolf-psort.html

4
http://wlab.ethz.ch/protter/start/

5
http://www.sbg.bio.ic.ac.uk/phyre2/html/page.cgi?id=index

6
http://gsds.cbi.pku.edu.cn

7
http://mg2c.iask.in/mg2c_v2.0/

8
http://bioinformatics.psb.ugent.be/webtools/plantcare/html/search_CARE.html

9
http://crispr.hzau.edu.cn/CRISPR2/

## Supplementary material

Supplementary Figure 1 The process of tobacco genetic transformation.

Supplementary Figure 2 The transmembrane helices analysis of NtLHT proteins.

Supplementary Figure 3 Predicted 3D structures of the NtLHT proteins.

Supplementary Figure 4 Expression levels of *NtLHT22* in wild-type plants and two overexpression plants.

Supplementary Figure 5 Construction and verification of *NtLHT22* knock out transgeneic plants. **(A)** Two separated target sites designed for knock out the *NtLHT22* by CRISPR/Cas9. **(B)** Verification of the knockout lines of *NtLHT22* by PCR-based sequencing.

Supplementary Table 1 The LHT protein sequences of tobacco, tea, *Arabidopsis*, and rice.

Supplementary Table 2 The *LHT* coding sequences of tobacco.

Supplementary Table 3 Primers used in this study.

Supplementary Table 4 Ka and Ks ratios for seven paralogous *NtLHT* genes.

Supplementary Table 5 Collinear *LHT* genes between tobacco, tea, *Arabidopsis*, and rice.
