# Supplementary figures and images for: Comprehensive analysis of the LHT gene family in tobacco and functional characterization of NtLHT22 involvement in amino acids homeostasis
Source: Front Plant Sci. 2022 Sep 13;13:927844. doi: 10.3389/fpls.2022.927844 (PMC9513474; doi:10.3389/fpls.2022.927844)

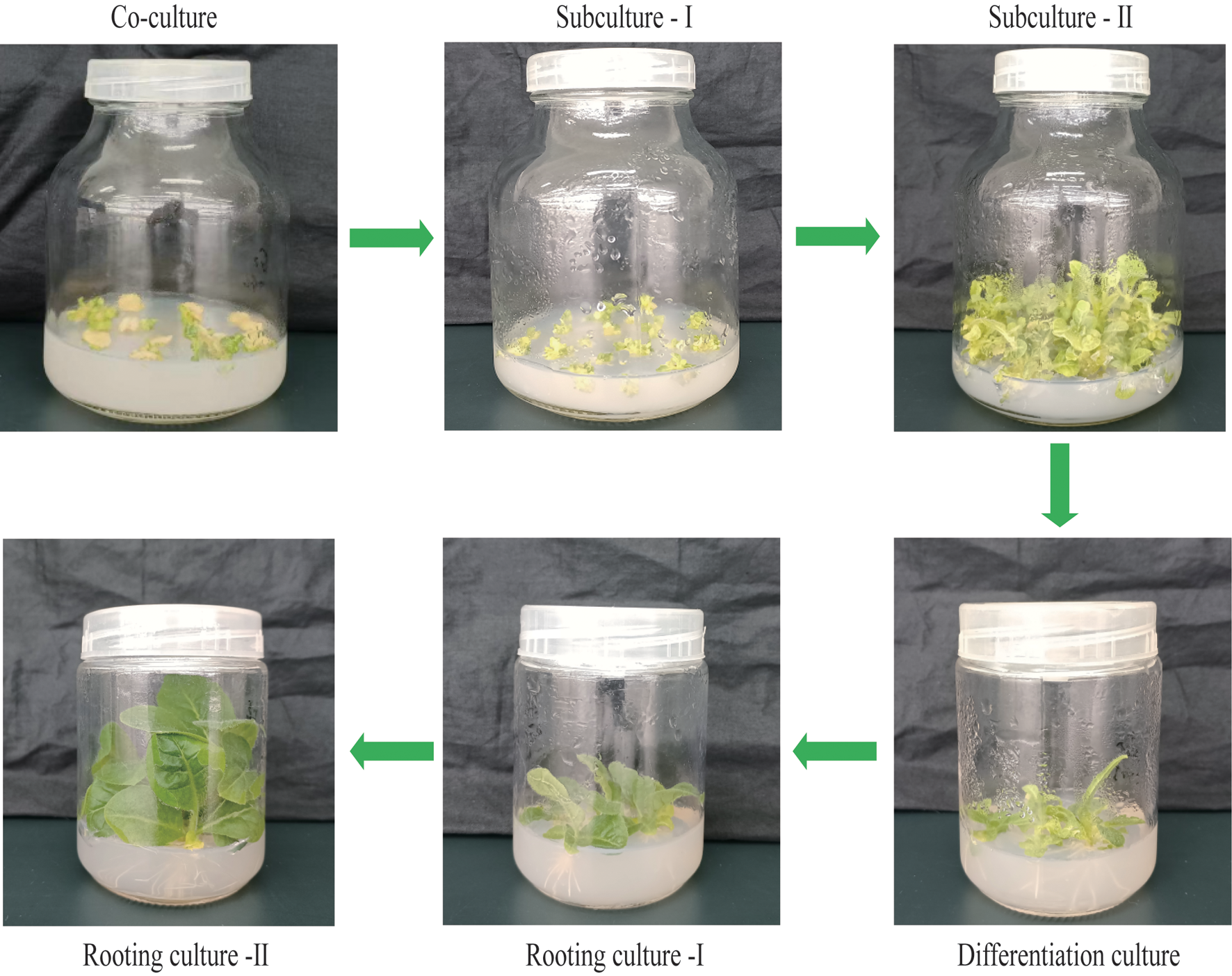

Supplement: Supplementary Figure 1 — The process of tobacco genetic transformation. [file Image_1.TIF]

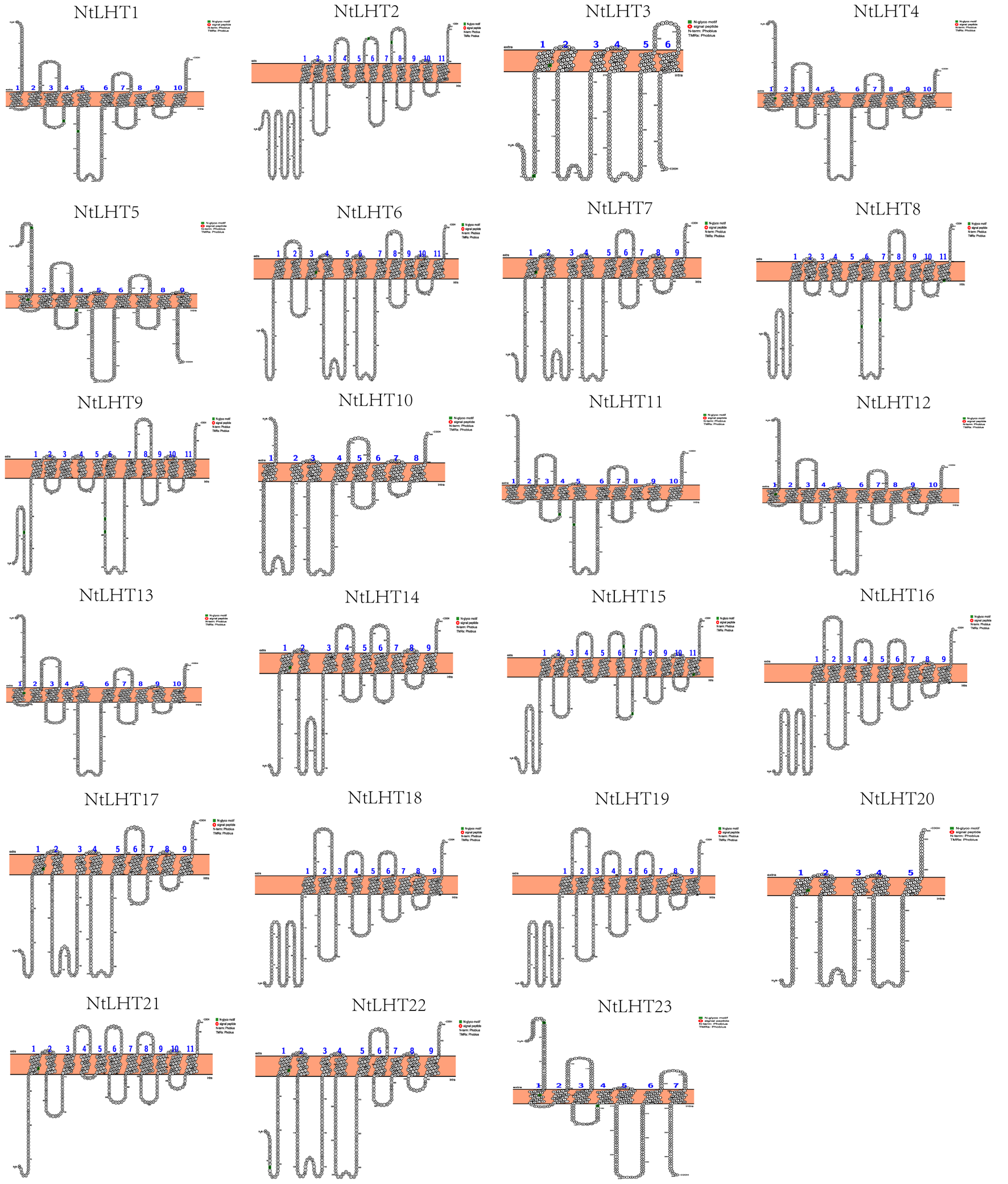

Supplement: Supplementary Figure 2 — The transmembrane helices analysis of NtLHT proteins. [file Image_2.TIF]

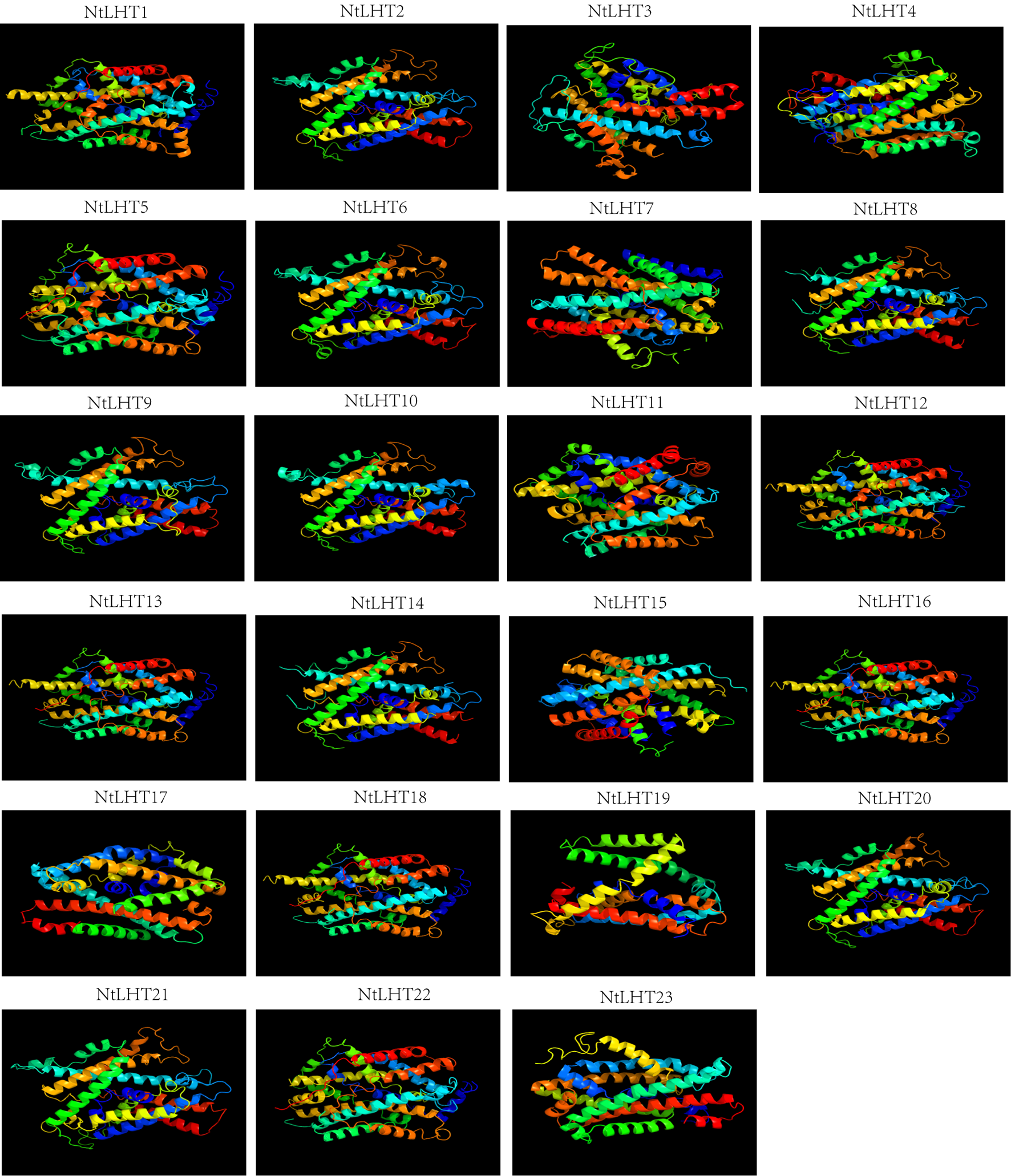

Supplement: Supplementary Figure 3 — Predicted 3D structures of the NtLHT proteins. [file Image_3.TIF]

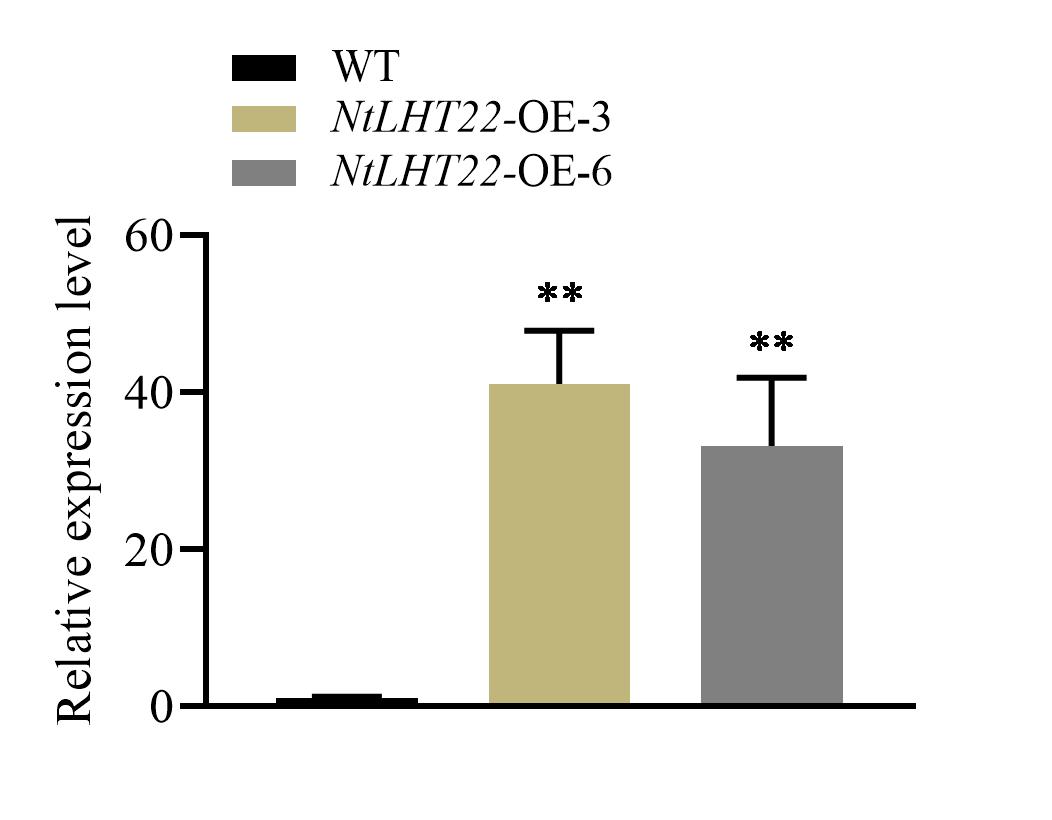

Supplement: Supplementary Figure 4 — Expression levels of NtLHT22 in wild-type plants and two overexpression plants. [file Image_4.JPEG]

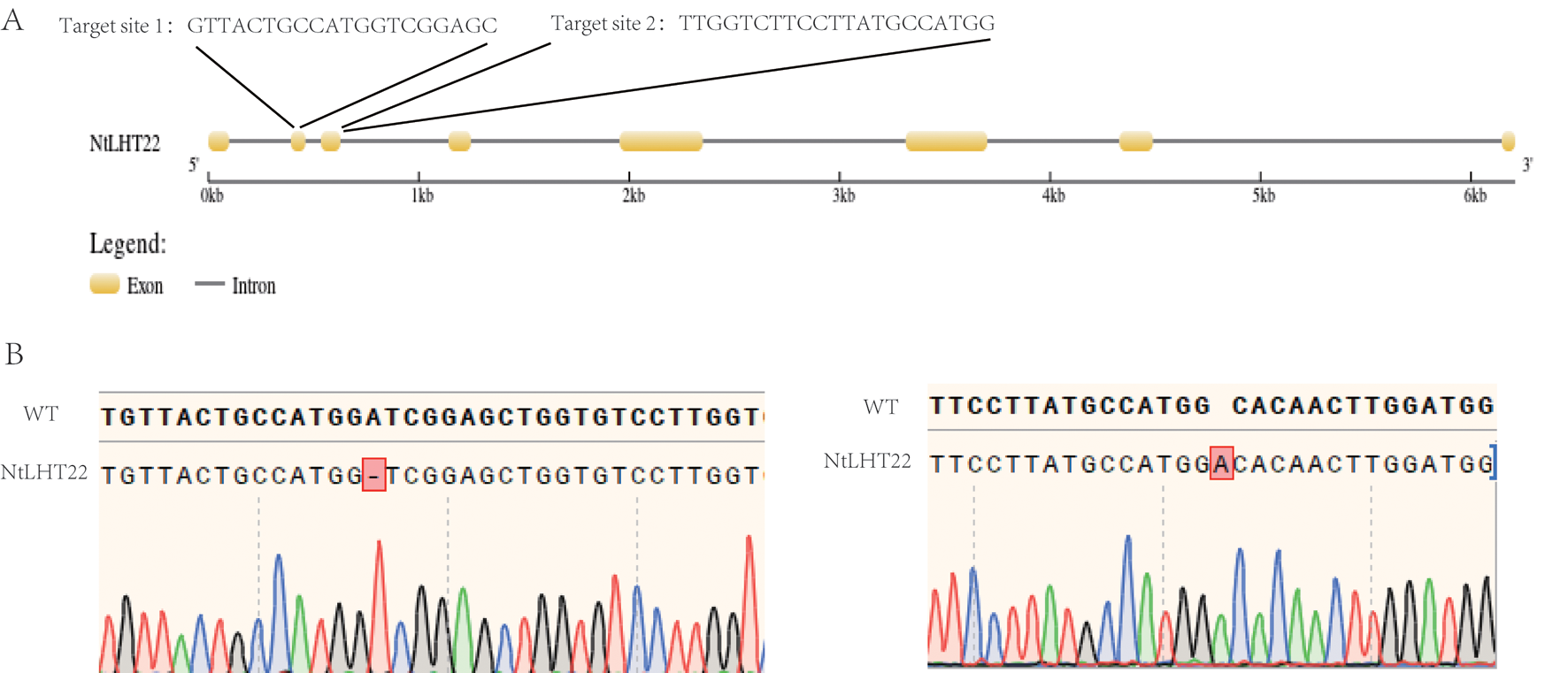

Supplement: Supplementary Figure 5 — Construction and verification of NtLHT22 knock out transgeneic plants. (A) Two separated target sites designed for knock out the NtLHT22 by CRISPR/Cas9. (B) Verification of the knockout lines of NtLHT22 by PCR-based sequencing. [file Image_5.TIF]
