# Supplementary material for: Comprehensive analysis of the LHT gene family in tobacco and functional characterization of NtLHT22 involvement in amino acids homeostasis
Source: Front Plant Sci. 2022 Sep 13;13:927844. doi: 10.3389/fpls.2022.927844 (PMC9513474; doi:10.3389/fpls.2022.927844)
Supplement: Supplementary Table 1 — The LHT protein sequences of tobacco, tea, Arabidopsis, and rice. [file Table_1.docx]

Table S1 The LHT protein sequences of *Arabidopsis*, rice, tea, and tobacco

***Arabidopsis thaliana***

AtLHT1

MVAQAPHDDHQDDEKLAAARQKEIEDWLPITSSRNAKWWYSAFHNVTAMVGAGVLGLPYAMSQLGWGPGIAVLVLSWVITLYTLWQMVEMHEMVPGKRFDRYHELGQHAFGEKLGLYIVVPQQLIVEIGVCIVYMVTGGKSLKKFHELVCDDCKPIKLTYFIMIFASVHFVLSHLPNFNSISGVSLAAAVMSLSYSTIAWASSASKGVQEDVQYGYKAKTTAGTVFNFFSGLGDVAFAYAGHNVVLEIQATIPSTPEKPSKGPMWRGVIVAYIVVALCYFPVALVGYYIFGNGVEDNILMSLKKPAWLIATANIFVVIHVIGSYQIYAMPVFDMMETLLVKKLNFRPTTTLRFFVRNFYVAATMFVGMTFPFFGGLLAFFGGFAFAPTTYFLPCVIWLAIYKPKKYSLSWWANWVCIVFGLFLMVLSPIGGLRTIVIQAKGYKFYS

AtLHT2

MGNSEMSASEVAAAKQKNVDDWLPITSSRNAKWWYSAFHNVTAMVGAGVLSLPYAMSNLGWGPGVTIMVMSWIITLYTLWQMVEMHEIVPGKRLDRYHELGQHAFGEKLGLWIVVPQQLIVEVGVDIVYMVTGGASLKKVHQLVCPDCKEIRTTFWIMIFASVHFVISHLPNFNSISIISLAAAVMSLTYSTIAWAASVHKGVHPDVDYSPRASTDVGKVFNFLNALGDVAFAYAGHNVVLEIQATIPSTPEMPSKVPMWRGVIVAYIVVAICYFPVAFLGYYIFGNSVDDNILITLEKPIWLIAMANMFVVIHVIGSYQIFAMPVFDMLETVLVKKMNFNPSFKLRFITRSLYVAFTMIVAICVPFFGGLLGFFGGFAFAPTTYYLPCIMWLVLKKPKRFGLSWTANWFCIIVGVLLTILAPIGGLRTIIINAKTYKFFS

AtLHT3

MKGIPSSSNQILNQDLVEDQSFELEDWLPITASRNANWYYSAFHNVTAIVGAGVLGLPYAMSELGWGPGVVVLILSWVITLYTFWQMIEMHEMFEGKRFDRYHELGQAAFGKKLGLYIVVPLQLLVETSACIVYMVTGGESLKKIHQLSVGDYECRKLKVRHFILIFASSQFVLSLLKNFNSISGVSLVAAVMSMSYSTIAWVASLTKGVANNVEYGYKRRNNTSVPLAFLGALGEMAFAYAGHNVVLEIQATIPSTPENPSKRPMWKGAIVAYIIVAFCYFPVALVGFWTFGNNVEENILKTLRGPKGLIIVANIFVIIHLMGSYQVYAMPVFDMIESVMIKKWHFSPTRVLRFTIRWTFVAATMGIAVALPHFSALLSFFGGFIFAPTTYFIPCIIWLILKKPKRFSLSWCINWICIILGVLVMIIAPIGGLAKLMNALKQPDSSCKST

AtLHT4

MDERPETELISIPATPRVSTPEILTPSGQRSPRPATKPSSATWTPTSFISPRFLSPIGTPMKRVLVNMKGYLEEVGHLTKLNPQDAWLPITESRNGNAHYAAFHNLNAGVGFQALVLPVAFAFLGWSWGILSLTIAYCWQLYTLWILVQLHEAVPGKRYNRYVELAQAAFGERLGVWLALFPTVYLSAGTATALILIGGETMKLFFQIVCGPLCTSNPLTTVEWYLVFTSLCIVLSQLPNLNSIAGLSLIGAVTAITYSTMVWVLSVSQPRPATISYEPLSMPSTSGSLFAVLNALGIIAFAFRGHNLVLEIQSTMPSTFKHPAHVPMWRGAKISYFLIALCIFPISIGGFWAYGNLMPSGGMLAALYAFHIHDIPRGLLATAFLLVVFSCLSSFQIYSMPAFDSFEAGYTSRTNKPCSIWVRSGFRVFFGFVSFFIGVALPFLSSLAGLLGGLTLPVTFAYPCFMWVLIKKPAKYSFNWYFHWGLGWLGVAFSLAFSIGGIWSMVTNGLKLKFFKPPN

AtLHT5

MEKSQSSPTKDASTKQKNVDDWLPITSSRNAKWWYSAFHNVTAMVGAGVLSLPYAMSNLGWGPGVTIMIMSWLITFYTLWQMVQMHEMVPGKRFDRYHELGQHAFGEKLGLWIVVPQQLIVEVGVDIVYMVTGGKSLKKIHDLLCTDCKNIRTTYWIMIFASIHFVLAHLPNFNSISIVSLAAAVMSLSYSTIAWATSVKKGVHPNVDYSSRASTTSGNVFNFLNALGDVAFAYAGHNVVLEIQATIPSTPEKPSKIAMWKGVVVAYIVVAICYFPVAFVCYYIFGNSVDDNILMTLEKPIWLIAIANAFVVVHVIGSYQIYAMPVFDMLETFLVKKMMFAPSFKLRFITRTLYVAFTMFVAICIPFFGGLLGFFGGFAFAPTTYYLPCIMWLCIKKPKKYGLSWCINWFCIVVGVILTILAPIGGLRTIIISAKNYEFFS

AtLHT6

MAGIPDHIQDQHLVEEDQPFDLEDWLPITASRNANWYYSAFHNVTAIVGAGVLGLPYAMSELGWGPGVVVLILSWVITLYTLWQMIEMHEMFEGQRFDRYHELGQAAFGKKLGLYIIVPLQLLVEISVCIVYMVTGGKSLKNVHDLALGDGDKCTKLRIQHFILIFASSQFVLSLLKNFNSISGVSLVAAVMSVSYSTIAWVASLRKGATTGSVEYGYRKRTTSVPLAFLSALGEMAFAYAGHNVVLEIQATIPSTPENPSKRPMWKGAVVAYIIVAFCYFPVALVGFKTFGNSVEESILESLTKPTALVIVANMFVVIHLLGSYQVYAMPVFDMIESVMIRIWHFSPTRVLRFTIRWTFVAATMGIAVGLPYYSALLSFFGGFVFAPTTYFIPCIMWLILKKPKRFSLSWCMNWFCIIFGLVLMIIAPIGGLAKLIYNIQKGTLPNSRCNLPKH

AtLHT7

MSIALGNLFDLESQESGGSPLFMSPAPSTDPQPISGEKNGGDGGRIPVEEWLPITESRKGNVYTATFHLLCSGIGLQVILLPAAFAALGWVWGTIILTVGFVWKLYTTWLLVQLHEAVPGIRISRYVRLAIASFGVKLGKLLGIFPVMYLSGGACTILVITGGKSIQQLLQIMSDDNTAPLTSVQCFLVFSCIAMIMSQFPNLNSLFGVSLIGAFMGIAYCTVIWILPVASDSQRTQVSVSYATMDKSFVHIFNAIGLIALVYRGNNLVLEIQGTLPSDSKNPSCKTMWRAVMISHALVAICMFPLTFAVYWAYGDKIPATGGPVGNYLKLYTQEHSKRAACFIHLTFIFSCLCSYPINLMPACDNIEMVYITKKKKPASIIVRMMLRVFLSLVCFTIAVGFPFLPYLAVLIGAIALLVTFTYPCFMWISIKKPQRKSPMWLFNVLVGCLGASLSVLLLVASAMRLAQKGLHANFFRP

AtLHT8

MTYTANDEENKGRSTDNNNHRQMDYNDWLPVTASREAKWYYSAFHNVTAMVGAGVLGLPFAMSQLGWGPGLVAIIMSWAITFYSLWQMVQLHEAVPGKRLDRYPELGQEAFGPKLGYWIVMPQQLLVQIASDIVYNVTGGKSLKKFVELLFPNLEHIRQTYYILGFAALQLVLSQSPDFNSIKIVSLLAALMSFLYSMIASVASIAKGTEHRPSTYGVRGDTVASMVFDAFNGIGTIAFAFAGHSVVLEIQATIPSTPEVPSKKPMWKGVVVAYIIVIICYLFVAISGYWAFGAHVEDDVLISLERPAWLIAAANFMVFIHVIGSYQVFAMIVFDTIESYLVKTLKFTPSTTLRLVARSTYVALICLVAVCIPFFGGLLGFFGGLVFSSTSYFLPCIIWLIMKRPKRFSAHWWCSWVAIVTGISIAILAPIGGMRHIILSARTYKLFS

AtLHT9

MVSSSPVSPSKETDRKSGEKWTAEDPSRPAKWWYSTFHTVTAMIGAGVLSLPYAMAYLGWGPGTFVLAMTWGLTLNTMWQMVQLHECVPGTRFDRYIDLGRYAFGPKLGPWIVLPQQLIVQVGCNIVYMVTGGKCLKQFVEITCSTCTPVRQSYWILGFGGVHFILSQLPNFNSVAGVSLAAAVMSLCYSTIAWGGSIAHGRVPDVSYDYKATNPGDFTFRVFNALGQISFAFAGHAVALEIQATMPSTPERPSKVPMWQGVIGAYVVNAVCYFPVALICYWAFGQDVDDNVLMNLQRPAWLIAAANLMVVVHVIGSYQVFAMPVFDLLERMMVNKFGFKHGVVLRFFTRTIYVAFTLFIGVSFPFFGDLLGFFGGFGFAPTSFFLPSIMWLIIKKPRRFSVTWFVNWISIIVGVFIMLASTIGGLRNIIADSSTYSFYA

AtLHT10

MYIQMTDGVPPPPEQSSLDHRIDELERQKEIDDWLPITSSRNAKWWYSTFHNVTAMVGAGVLGLPFFMAQLGWGPGIAVLILSWIITLYTLWQMVEMHEMVPGKRFDRYHELGQFAFGERLGLYIIVPQQIIVEVGVCIVYMVTGGQSLKKFHEIACQDCSPIRLSFFIMIFASSHFVLSHLPNFNSISGVSLVAAVMSLSYSTIAWTATAAKGVQEDVQYGYKSGTTASTVLSFFTGLGGIAFAYAGHNVVLEIQATIPSTPSNPSKGPMWRGVVVAYVVVALCYFPVALVGYGVFGNAVLDNVLMSLETPVWAIATANLFVVMHVIGSYQIFAMPVFDMVETFLVKKLNFKPSTVLRFIVRNVYVALTMFIGIMIPFFGGLLAFFGGFAFAPTSYFLPCIMWLLIYKPKRFSLSWWTNWVCIVLGVVLMILSSIGGLRQIIIQSKDYSFFS

***Oryza sativa***

OsLHT1

MGTQVADNYPPAKDGRSAQEKAIDDWLPITSSRNAKWWYSAFHNVTAMVGAGVLSLPYAMSELGWGPGIAVLILSWIITLYTLWQMVEMHEMVPGKRFDRYHELGQHAFGEKLGLWIVVPQQLVVEVGVNIVYMVTGGKSLKKFHDVLCEGHGCKNIKLTYFIMIFASVHFVLSQLPNFNSISGVSLAAAVMSLSYSTIAWGASVDKGKVADVDYHLRATTSTGKVFGFFSALGDVAFAYAGHNVVLEIQATIPSTPEKPSKKPMWKGVVVAYIIVALCYFPVALVGYWAFGNHVDDNILITLSRPKWLIALANMMVVIHVIGSYQIYAMPVFDMIETVLVKKLRFPPGLTLRLIARTLYVAFTMFIAITFPFFGGLLGFFGGFAFAPTTYFLPCIMWLAIYKPRRFSLSWFTNWICIILGVMLMILSPIGGLRQIIIDAKTYKFYS

OsLHT2

MAPPSAMNTSRAEEKAIDDWLPITSSRNAKWWYSAFHNVTAMVGAGVLSLPFAMSELGWGPGVAAMIMSWVITLYTLWQMVEMHECVPGRRFDRYHELGQHAFGDKLGLWIVVPQQLVVEVGVCIVYMVTGGKSLKKFHDLVAPPSAPPIRTSYFIVIFGCLHLVLSQLPNFNSISGVSLAAAVMSLSYSTIAWAASLHHHNHNNGAAAGGVDYSLTEATPAGRTFNFLSALGDVAFAYAGHNVVLEIQATIPSTAERPSKGPMWRGVVLAYGVVAVCYLPVAFAGYYVFGNAVDDNVLITLERPAWLIAAANMFVVVHVVGSYQIYAMPVFDMLETFLVKKLRFKPGMPLRLIARSLYVLFTMFVAIAVPFFGGLLGFFGGFAFAPTTYFLPCIMWLSIMKPKRFGLSWCINWFCIIIGVLLSVFAPIGGLRSIIVNAQSYKFFS

OsLHT3

MSATEVMEECTETARERREEERLRNVNLDDWLPITSSRTAKWYYSAFHNVTAMVGAGVLGLPFAMSQLGWPTGVAAIASSFAITLYTLWQLVELHEPAPGGGKRFDRYHELGQAAFGRRLGVCLIVPLQLIVQVGTDIVYMVTGGQTLKKFVELACDGRCADIRLTFYIMMFASAQFVLSQCPNFNSISAVSAAAAAMSLCYSMIAFFASVLKAHPAAAAAVDYGFKATTAAGRVFGAFNALGAVSFAFAGHNVVLEIQATIPSTPERPSKRPMWRGVVVAYAVVALCYFTVAFGGYHAFGNAVAPNVLISLEKPRWLVAAANLMVVVHVIGAYQVYAMPVFDMIETVLAKKLHLRPGLPLRVTARSAYVALTMFIGITFPFFDGLLGFFGGFGFAPTTYFIPCIIWLIMRKPAKYSLSWLMNWCFIIIGMLLMLVSPIGGLRQIILDASKYKFYS

OsLHT4

MVTSSVLPKVVDDAGEGEANPRRAKWWYATFHSVTAMVGAGVLSLPYAMAHLGWGPGTAALVVSWGMTLYTLRLLIELHECVPGVRFDRYRDLGAHALGPRLGPWLVVPQQLIVQLGCDVVYMVIGGKCLMKFAESVSSWSRAPQLHHQSYWICIFGASQFLLSQLPSLDSITAVSLAAAAMSVGYSTISWAACLARGTPAAAEGGGGGVSYAYKDGTAADSVFRVCSALGQVAFAYAGHGVVLEIQATIPSTPTKPSRGAMWKGAVAAYLVTALCYFPVAIAGYWAFGRDVSDNVLVALRRPPWLVAAANMMVVVHVLGSYQVYAMPIFETLETILITRIRLPPGALLRLVARSAYVAFTLFVAVTFPFFGDLLGFFGGFGFTPTSYFLPCILWLKIKKPPRFSASWFANWGCIVVGVLLMIASTIGGLRSIIQDASTFQFYS

OsLHT5

MSSEVTSVPPTPTPPPVSTPPSQIQSPAAPASSRASPLRGMGTPNIASPVRKAVASVSGYLGEVGQMTRLADPRDAWLPITESRSGNAYYAAFHSLSSGIGFQALVLPVAFSLLGWTWAIICLTVAFAWQLYTLWLLVKLHEPVAGGTRYSRYMYLATTVFGEKWGKILALLPVMYLSAGTCTALIIVGGGSMKLLFNIACGEVCLARPLTTVEWYLVFVCVAALLSQLPNLNSIAGVSLVGATAAVAYCTMIWVVSVAKGRVAGVSYDPVRTTDEEDGAIGILNGLGIIAFAFRGHNLVLEIQATMPSTLKHPSHVPMWKGVKAAYVIIALCLYPVAVGGFWAYGDQIPPNGILSALYKFHSQDVSRVVLGTATLLVIVNCLTTYQIYAMPVFDNMETGYVHKKNRPCPWWMRAGFRALFGAINLLIAVALPFLSELAGLLGGISLPVTLAYPCFMWVAIMRPAKGTAMWYTNWGLGSLGMGLSFVLIVGNLWGLVEKGLHVKFFKPADFQ

OsLHT6

MAMAVEGAGAGVVVSEPEVVSIPPTPRGMSTPDGTATPPPAAGRGAATPARRVVEGLRGYLEEVGHLTRLNPQDAWLPITESRSGNARYAAFHTLNAGLGFQALLLPLAFPALGWSWGIISLTVAYFWQLYTLWILVKLHEAVPGRRYNRYVELAQAAFGEKLGMWLSLFPTIYLSAGSATALILVGGETMKLFYQIVCGPICSPSPISTIEWYLVFTSLAIVLSQLPNLNSIAGVSLIGGTTAITYCTMSWVLSVSQPQPPTVSYEPQAYTSLGSSLFSTLNALGIIAFAFRGHNLALEIQATMPSTFKHPAHVPMWRGAKVAYALIAMCLFPVAIGGYWAYGNMVPQGGMLTALYAFHSHDIPRGLLAATFLLVVFNCLSSFQIYSMPVFDSFEAAYTGRTNRPCSVWVRSGFRVFYGFLSLFISVALPFLSSLAGLLGGLTLPVTFAYPCFMWICIKKPERFSSGWYLNWGLGLLGTAFSLALCVGGVWSIISSGMKFKFFKPPS

***Camellia sinensis***

CsLHT1

MGKTPVRMKAVVYALSPFQQKVMPGLWNIVASLPVADNKKLITTGMVLHAGVLFSDCWRRCYIGDRCGAAIVPMKDESAGEVSVACVVRTNGFEVTEDEIKKYISEQVDTRSEKEKAIDAWLPITSSRNAKWWYSAFHNVTAMVGAGVLGLPYAMAELGWGPGVSALVISWVVTLYTLWQMVEMHEIVPGKRFDRYHELGQHAFGEKLGLYIVVPQQLIVEISVNILYMVTRGKSLKKFHDVVCKGCSNIKLTYFIMIFSSVHFVLSHLPNFNSISGVSLAAAVMSLSYSTIAWAASLHKGVQPHVEYGYKAKTTVGTVFNFFSALGEVAFAYAGHNVVLEIQATIPSTPEKPSKGPMWKGVIVAYIVVALCYFLVSLIGYWVFGNKVDDDILITLEKPAWLIAMANLFVVIHIYAMPVF

DMIETALVKKLHFKPTMMLRFITRNIYVALTMFVAITFPFFSGLLGFFGGFAFAPTTYFPPCVMWLASKKPEKFSLSWLINWTGLIKWETSKAKKIVPYKKMWLEKSQMIMHTALFGISMMSIMWCSKDNKGN

CsLHT2

MGEVDEVRSAPITPRPPSMASTPPISCPPSQFHSPSLSRSPLLSTADHPETTANKTPNKTPRIRTPRFITPLGSPLRKALKMTKLDPQDAWLPITESRNGNAYYAAFHTLCSGIGIQALVLPVAFTILGWTWGIILLTLGFVWQLYTLYLLVQLHESTETGIRYSRYMQISSATFGDKLAKLLALFPIAYLSGGTCVALIIIGGSTMRSLFQLACGANCSLKPLTIVEWYLVFTCAAVLLSQLPNLNSIAGVSLIGAITAVGYCTIIWVVSVAKGRLPNVSYEPVRSHSQMVNAFDLLNAIGIIAFAFRGHNLTLEIQATMPSSEKHPSRVPMWKGVKFAYAVVAMCLFPIAIGGYWAYGRQV

CsLHT3

MYLSGGTCVTLIIIGGSTANIFFQLACGATCSMKPLTAVEWYLVFTCAVVVLCQLPNLNSIAGVSLVGAITAVGYCTSMWVVSLVKGRLPGVSYHPVSASNDVAKFFLVLNGLGIIAFAFRGHNLILEIQGTMPSSEKYPSHVPMWKGVKVAYLLIALCLFPLAIAGYWAYGHLIPNGGMLSALFQFHSRDLSKPVLELICLFIIINALSSFQIYGMPVIDHMESHYTSRMKKPCSWWIRSLIRAMFTFGSFFFAVAIPFLGSLAGLLGGIALPVTLAFPCFMWLKMKKPKKYSGMWWLNWGLGLLGVGLTVFIIAGGVYVVITTGIKVSFFKPAV

CsLHT4

MDERPETELICSIPATPRASTPETQTPSGQRSPRPASKASTGWTPTSLLSPIGTPMKRVLVNMKAYLEEVGHLTKLNPQDAWLPITESRNGNAHYSAFHNLNAGVGFQALVLPVAFSFLGWSWGILSLTIAYIWQLYTLWILVQLHEAVPGKRYNRYVELAQAAFGERLGVWLALFPTVYLSAGTATALILIGGETMKLFFQIVCGPLCSSNPLTTVEWYLVFTFLCIVLSQLPNLNSIAGLSLIGAITAITYSTMVWVLSVSQQRPPSISYHPLSLPSFSASVFSVMNALGIVAFAFRGHNLALEIQATMPSTFKHPAHVPMWRGAKVAYFFIAMCLFPVAIGGFWAYGNLMPSGGILNALFAFHNHDIPRGLLAMTFLLVVFNCLSSFQIYSMPVFDSFEAGYTSRTNRPCSIWVRSGFRVFYGFISFFIGVALPFLSSLAGLLGGLTLPVTFAYPCFMWVLIKKPTKFSFNWYLNWILGWLGIAFSLAFTIGGIWSMVNNGLTLKFFKPN

CsLHT5

MDVVVEVNSIPLPETKTPVISAPPFQLHSPSLTRSPLLDSIIPKTPKSPLVRMMTPMASPMKKAFSTMQGYLEEVGNLTKLNPQESWLPITESRNGNAYYAAFHSLNSGIGVQALLLPLAFTALGWTWGIICLSVAFVWQLYTLWLLIQLHESVPGTRYSRYLQLSMAAFGKKIGKLVVLFPTMYLSGGTCVALIMIGGGTMKILFQIVCGSTGSSCNANSLTTVEWYIVFTTSAVILAQLPNLNSIAGVSLIGAITAVAYCTLIWTLSVVKGRPIGFSYGPSQVAESDGAKLYTTLDALGMIAFAFRGHNLVLEIQGTMPSSLKHPSRLPMWKGVKFSYLIIALCLFPVAIGGYWAYGNLISNGGMLKALYKYHGHDTSKIILGFASVLVVVNSLTSFQIYAMPVFDNLEFRYTSNMNRPCPWWLRRGFRVFFGCLAFFIAVALPFLPSLAGLIGGIALPVTAAYPCFMWIIIKKPQKYGTMWCLNCALGCLGVILSILLVFGAIWTIVALGIEVHFFKP

CsLHT6

MVSNSPPAPKEVPTEEDQWMEKGPPREAKWWYSTFHTVTAMVGAGVLSLPYAMAYLGWGPGTMLLVFSWCITLNTMWQMIQLHECVPGVRFDHYYDLGRHAFGPKLGPWIVLPQQLIVQVGCDIVYMVTGGKCLKKFMEIVCTNCTRIKQSYWICIFGSTHFFLSQLPNFNSVSGVSLAAAIMSLSYSTIAWVGCLSKGRVENVSYAYKKTSGADSMFRVFNALGEITFAYAGHAVVLEIQATVPSTPEMPSKVPMWKGAVWAYFINAICYFPVALIGYWAFGQDVADNVLVALERPSWLIAAANLMVVIHVIGSYQVYAMPVFDLLEKAMVKRLSFPPGIGLRIIVRSAYVAFTLFVGVTFPFFGDLLGFFGGFGFAPTSYFLPSIIWLIIKKPKRFSISWFINWLCNFARQLASWRAKSARQGTPKLASEVSSATCFLKSKISLARNFNIEEQSQLDNLPLGEKKASSTTCFLASKISSARNSKVGEEGQLDNLLLSEQNQFGKEFLSWNAKSARQLASWRAKSTRQGTPKLASKVCSATCFLVRKISSVKNFKVGEQGQLGNLLLGKQNQLAKTSWRVKLAWQGILKLIKTINKSLAEQSSPQQGIPKLIKTPIFILPSKISSARNFDLFVKMIVSYLLDSITSTW

CsLHT7

MGEVGEVCVIEAANHHQIAKPTKEKRQMIQLIPATATATATVMASPDDRDIEATTMLANLGGSISHETEHNNPLEAWLPITESRDGNAFTSAFHVLCSGIGVQALLLPLAFTSLGWLWGIVCLSMAFGWQLYTIWVLVHLHESAPGTRYSRYLHLAIAAFGAKLGKLLAIFPVMYLSGGTCVMLIITGAKTMELFYNTICGDGLTCIARTPSGAEWFLVFTFLAIFVAQFFPNLNSIAGVSLIGAITAVGYCTLLWALSISLDRPDGASHDPSKAASSETARTRSVLTALGIIALAFRGHNLVLEIQGTMPSSSKHPSREPMWRGVTMSYILISMCLFPLAIGGYWAYGNSIPADGGMLITFSKFHGQSTSKFVVGLIYLLVLINCLCSFQIYAMPVFDNLEFQYTIKQETRCPRMAEPHPKDHGSEAVKLPVETMDSGMFDCFGKKEEKPGEDVAMIVLETVQVSETDKMKEKHTLMEELHWSHSNSSSSSDDKEGGEKKRKKGLKEKLKKKMSSEKEEQVNEHKNTSGSLENCNEVGNAEATQQEEKKGFLEKIKEKLPGQHKKAEEGSPAAPPPECAADGNSPGNETKDKKGILEKIKEKLPGYHKNDDDNVKEKEN

***Nicotiana tabacum***

NtLHT1

MVSSSPPPAPKEVPSDEKWAEDGPPREAKWWYSTFHTVTAMVGAGVLSLPYAMAYLGWGPGTAVMILSWCITLHTMWQMIQLHECVPGVRFDRYKDLGKHAFGPKLGAWIVLPQQLIVQVGCDIVYMVTGGKCLKKFMEIACTNCTTIRQSYWICIFGAIHFFLSQLPNFNSVSGVSLAAAVMSLSYSTIAWVGCIGKGRVPNVSYAYKKTSPADSMFRVFNALGQVSFAYAGHAVVLEIQATIPSTPEKPSKVPMWKGAVWAYFVNALCYFPVAFIGYWAFGQDVDDNVLVGLERPSWLIAAANLMVVVHVIGSYQVYAMPVFDLMEQKMVKTWNFPPGVMLRFFVRTAYVAFTLFLGVTFPFFGDLLGFFGGFGFAPTSYFLPCIMWLKIKKPRRFSMSWLINWACIFIGVFIMIASTVGGLRNIVADSSTYEFYS

NtLHT2

MTNLDEASSLPSSLPITPRTVTVAPTFHFDDQFGSLPITPRTASVAQTPSVVSLPITPRTASVAQTPSIVSLPPSQFHSPSLSRSPLLNVGDHATSAANRASKTPRSRGLTPRFITPLGSPLRKALKMTRLDPQDAWLPITESRNGNAYYAAFHTLCSGIGIQALVLPVAFTILGWAWGVISLTAAFVWQLYTLYLMVQLHENYETGIRYSRYLQLACATFGDKLGKLCAAFPIGYLSAGTCCALIIIGGSTAKLLYQTLCGATCSNPKPLTTVEWYLVFTCVAVVLAQLPNLNSIAGISLVGALTAVGYCTALWTVSVAEGRLPNVSYDPVRKGTQVARIFDLLNALGIIAFAFRGHNLILEIQATMPSSEKHPSRVPMWRGVQFSYLLIAMCLFPLAIGGYWAYGHLIPANGSMLTALFAFHSQDVSRSVLALISIFVIINAVSSFQIYGMPMFDDMESAYTTRSKKACPWWLRSIFRAIFGFVCFFIAVAIPFLGSFAGLIGGIALPVTFAYPCFMWLKVKKPHKYSLSWWVNWGLGLLGMGLSGILVAAGLYVVIDTGVKISFFNPQ

NtLHT3

MHDKEVSRVESGNNSNDATANEQIDDDLNRWLPITASRKAKWWYSTFHNVTAVVGAGVLGLPYAVSQLGWIPGIGMIIISWFVTLYSLWQLVNLHEHVPGKRFDRYPELGKHVFGLKRGYWIVMPQQMIVQVASDIVYMVTGGKSLKESMHTMFHWSRGIKQTYFILFFGVLQLILSQAPNFNSLKVVSFTAAVMSLSYSTISSIASIIKGVEHPQPVNYGLRSHTPVGITFDIFNSLGTIAFAFAGHSVALEIQATIPSTPEKPSKGPMWRGVTVAYAIVAFCYLAVAASGFWAFGNLVDDDVLVTLKHPHWLIALANFMVFLHVLGSYQVFAMPVFDMIECYLVKKRRFTPGRPLRLIARSIYVGK

NtLHT4

MAHANEKEKDTRTEEEKAIDAWLPITSDRNAKWWYSTFHNVTAMVGAGVLSLPYAMSEMGWGPGVTVMLLSWAITFYTIWQMVEMHEMVHGKRFDRYHELGQHAFGEKLGLWIVVPQQIVVDVSSCIIYMVTGGKSLKKFHETVCPDCQPIKLTYFIIIFSSVHFVLSHLPNFNSISLVSLAAAVMSLTYSIVAWAASVGRGIEGREVSYELRCEKTSDNIFMFLSALGDVAFAYAGHNVVLEIQATIPSTPEKPSKGPMWKGVWVAYLIVAVCYLPVAFIGYWAFGNVVEDNILLSLEKPTWVIAAANLFVVVHVIGSYQVFAMPVFDMIETYAVKSMRLKPSTILRFSVRTTYVALTLFVGLTIPFFGGLMGFFGGFALAPTSYYLPCIIWLIIVKPKRFGFSWFINWFCIIVGILLTVLSPIGGMWTLIKQAKNYRFYQ

NtLHT5

MVQSKEVSMVESGNNSDDGRTNEQIDDLNKWLPITASRKAKWWYSAFHNVTAIVGAGVLGLPCAVSQLGWIPGIGMIIVSWSVTLYSFWQLVNLHEHVPGKRFDRYPELGKHVFGVKRGYWMVMPQQMIVQVACDIVYMVTGGKSLRESVIMMFHWGRRINQTYYIMFFGVLQLILSQAPNFNSLKVVSFTAAVMSLSYSTIASIASIIKGIEHPKSANYGLRSHTTAGIIFDIFNSLGTIAFAFAGHSVALEIQATIPSTPERPSKGPMWRGVVVAYAIVAFCYLSVAASGFWAFGNLVADDVLVTLEHPNWLISLANFMVFLHVLGSYQVFAMPVFDTIESFLVKKRHFTPGRPLRLIARSVYVVLTMFVGICIPFFGGLLGFFGGLAFSSTSFFLPCMMWLVSHKPKRWSFHWTASWVSLSNSLR

NtLHT6

MGAVFNFFSALGDVAFAYAGHNVVLEIQATIPSTSEKPSKGPMWRGVVVAYIVVAVCYFPVALIGYWVFGNSVQDNILISLEKPTWLIVMANFFVVVHVIGSYQADGRTEEQKAIDEWLPITSSRNAKWWYSAFHNVTAMVGAGVLGLPYAMSELGWGPGVTVMVVSWVITLYTLWQMVEMHEMVPGKRFDRYHELGQHVFGNKLGLWIVVPQQLVVEVGLDIVYMVTGGKSFQKIHDLVCKDNCKDIKLTYYIMIFASVHFVLSHLPNFNAISGVSLVAAIMSLSYCTIAWGASIDKGVQPDVEYEYRAENAGEGVFNFFSGLGEVAFAYAGHNVVLEIQATIPSTPEKPSKGPMWKGVLVAYIIVALCYFPVAIIGYWIFGNSVSDNILISLEKPTWLIVLANAFVVIHIIGSYQLYAIPVFDMLETYLIKKLRFKPTWYLRFITRNLYVAFTMFVGIIFPFFGGLLGFFGGFAFAPTTYFLPCIMWLSIYKPKRWGLSWTTNWICIIVGVMLTVLAPIGGLRTIIMQAKDYKFFS

NtLHT7

MGTQAPSDPNYNNDKVDTRTAEEKAIDAWLPITSSRNAKWWYSAFHNVTAMVGAGVLSLPYAMAELGWGPGVTVMVVSWIITLYTLWQMVEMHEMVPGKRFDRYHELGQHAFGEKLGLWIVVPQQLIVEVGVDIVYMVTGGRSLMKVHELVCTKNEDNIHCTKDIKLSYFIMIFASVHFVLSHLPNFNSISGVSLAAAVMSLSYSTIAWGASVKKGVQPDVDYGYKAHSTSGTVFNFLSGLGEVAFAYAGHNVVLEIQATIPSTPEKPSKIPMWRGVVVAYIVVALCYFPVAFIGYWMFGNSVEDNILVSLNKPTWLIAMANMFVVVHVIGSYQIYAMPVFDMLETVLVKKLRFSPTWYLRFVTRNIYVAFTMFVGITFPFFGGLLGFFGGFAFAPTTYFLPCIMWLAIYKPRRWSLSWIANWICIIFGVLLMVLAPIGGLRSIIVQAKTYKFYN

NtLHT8

MGSELVEIKMSQSPLKIGDKEVQNTVSLTPSPILDSIPKTPKSPFGARIMTPLASPMKKALTYMEEIGHFTKLDPQDAWLPITESRNGNAYYAAFHTLSSGIGVQALVLPLAFITLGWIWGIISLSIIFMWQLYTLWLLIQLHESVPGMRYSRYLRLSMAAFGEKLGKILALFPTMYLSGGTCVTLIMIGGGTMKIFFQTICGSNHCHLTSLSTIEWYIVFTVSAIILAQLPNLNSIAGISLVGSISAVTYCTLTWVVSVVKERPEDVSFETVENKSDLERVCSILNAIGMIAFAFRGHNLVLEIQGTMPSSLKNPSHVPMWKGVKFSYSIIALCLFPLAIGGYWAYGNLMPNGGILSALDKYHGKDTSKVILGITSLLVVVHSLTSFQIYAMPVFDNLEFRYTSNKKKPCPWWLRTGFRVFFGCLAFFISVALPFLPSLAGLIGGIALPVTLAYPCLMWIMIKKPQTYTSTWFVNWSLGLLGLVLSVLLVFGAIWTIAIQGMDVHFFKPQ

NtLHT9

MGDIERVSSSFSSLKIIPIDNDDRFDNNQSEGRDSPSCMAAVDGGMEKKNMNIPEEEVESYLPITESRKGNAYTAAFHLLCSGIGTPALVLPFAFTSLGWSWGIIILTVVFAWRLYTMWLLVHLHESNSGTRYSRYLQLSIAAFGLKLGKCLAIFPIMYLSGGTCVMSIIAGGGTLQLFYNAICGNDHNCHHRSLSGVQWFLLFICLAILIAQFCPNLHSLSWVSFVGSVMGVAYLTLIWALSISKGRPNGVSYNPSDNATTTMARFRAILNGVAIIVIAFRGHNVVLEIQGTLPTNPKHPTRTSMWRGVVSSYSFIAMCIFPLAIGGYWSYGNLMPASGIMTAIAKYHQESTPKWLTGTIYIMVIIQSLCTFQIYAMPVFDNFERIYVSKQHKACPRWVKLCIKLFFGGLTYFISVAFPFLGSLAAFVGGIALPLSLVYPCFMWISIKKPSRNSLMYCLNMILGCLGMLISIVQVAGALWNLVVEKFDANFFSP

NtLHT10

MVGAGVLGLPYAMSELGWGPGVTVMVVSWVITLYTLWQMVEMHEMVPGKRFDRYHELGQHVFGNKLGLWIVVPQQLVVEVGLDIVYMVTGGKSFQKIHDLVCKDNCKDIKLTYYIMIFASVHFVLSHLPNFNAISGVSLVAAIMSLSYCTIAWGASIDKGVQPDVEYEYRAENAGEGVFNFFSGLGEVAFAYAGHNVVLEIQATIPSTPEKPSKGPMWKGVLVAYIIVALCYFPVAIIGYWIFGNSVSDNILISLEKPTWLIVLANAFVVIHIIGSYQLYAIPVFDMLETYLIKKLRFKPTWYLRFITRNLYVAFTMFVGIIFPFFGGLLGFFGGFAFAPTTYFLPCIMWLSIYKPKRWGLSWTTNWICIIVGVMLTVLAPIGGLRTIIMQAKDYKFFS

NtLHT11

MAHANEKEIDTRTEEEKAIDAWLPITSDRNAKWWYSTFHNVTAMVGAGVLSLPYAMSEMGWGPGITVMLLSWAITFYTIWQMVEMHEMVPGKRFDRYHELGQHAFGEKLGLWIVVPQQIVVDVSSCIIYMVTGGKSLKKFHETVCPDCQPIKLTYFIIIFSSVHFVLSHLPNFNSISLVSLAAAVMSLTYSIVAWACSIGRGIEGREVSYELRGEKTSDNIFMFLSALGDVAFAYAGHNVVLEIQATIPSTPEKPSKGPMWKGVWVAYLIVAVCYLPVAFIGYWAFGNVVEDNILLSLEKPVWVVAAANLFVVVHVIGSYQVFAMPVFDMIETYAVKSMRLKPSTLLRFGVRTTFVAFTLFVGMTIPFFGGLMGFFGGFALAPTSYYLPCIIWLIIVKPKRFGFSWFMNWFCIIAGILLTVLSPIGGMWTLIKQAKNYRFYQ

NtLHT12

MVSSSPPPAPKEVPSDEKWAEDGPPREAKWWYSTFHTVTAMVGAGVLSLPYAMAYLGWGPGTVVMILSWCITLHTMWQMIQLHECVPGVRFDRYKDLGKHAFGPKLGAWIVLPQQLIVQVGCDIVYMVTGGKCLKKFMEIACTNCTTIRQSYWICIFGAIHFFLSQLPNFNSVSGVSLAAAVMSLSYSTIAWVGCVGKGRVPNVSYAYKKTSPADSMFRVFNALGQVSFAYAGHAVVLEIQATIPSTPEKPSKVPMWKGAVWAYFVNALCYFPVAFIGYWAFGQDVDDNVLVGLERPSWLIAAANLMVVVHVIGSYQVYAMPVFDLMEQKMVKTWNFPPGIMLRFFVRTAYVAFTLFLGVTFPFFGDLLGFFGGFGFAPTSYFLPCIMWLKIKKPRRFSMSWLINWACIFIGVFIMIASTIGGLRNIVADSSTYEFYS

NtLHT13

MEKGGDNEDKRREEEKAIDEWLPITSDRNAKWWYSTMHNVTAMVGAGVLSLPYAMSQMGWGAGVTVLLLSWIITFYTIWQMVEMHEMIPGKRFDRYHELGQYAFGEKLGLWIVVPQQIVVEVSTCIIYMVTGGKSLKKFQEILFPNAKPIKLTYFILIFSSFQFILSHLPNFNSISSVSFVAAILSMTYSAIAWTVSLKESGREVSYGPTSEKTSDNVFMFLSALGNVAFAYAGHNVVLEIQATIPSTEDAPSKKAMWKGVLTAYIIVALCYLPVAFIGYWGFGNGVDDNILLTLHTPTWLIATANIFVVAHVIGSYQVYAMPVFDMIETYAVKSLRYKPSTILRVCVRTVFVAFTLVVGMTIPFFGGLMGFFGGFALAPTSYYLPCIIWLIIKKPRRFGLSWCTNWLCIIVGVLLTLTSPIGGLWSIIKSAKTYRFYT

NtLHT14

MTGEEKKKRGSGSEGSSNKDLNDWLPITSSRNAKWYYSAFHNVTAMVGAGVLGLPYAMSQLGWGAGATVMVLSWVITLYTLWQMVEMHEMVPGKRFDRYHELGQHAFGEKLGLWIVVPQQLMVEVGVNIVYMVTGGKSIKKIYDTACPSCRPLKTTYFIMMFSSIHFFLSHCPNFNSITLVSFLAAIMSLSYSTIGWGASVHKGISPEVDYSPRASTTTGRVFGFLSALGDVAFAFAGHNVVLEIQATMPSSPEKPAKKPMWKGVIFAYIVVALCYFPVAFAGYAVFGKSVEDNVLISLEKPAWLIIIANAFVVVHVIGSYQVFAMGVFDMVESYLVKQRKFTPTKTLRFIVRTSYVALTMFLGITFPFFGGLLGFFGGFAFAPTTYFLPCIMWLAIYKPKKFGLSWFTNWICIILGVLLMILAPIGALRQIILQAKDYKFYS

NtLHT15

MSQSTLKIGSEEVQNTVSLTPSPNLDSIPKTPKSPFGTRIMTPLASPMKKALTYMEEIGHFTKLDPQDAWLPITESRNGNAYYAAFHTLSSGIGVQALVLPLAFITLGWIWGIISLSIIFMWQLYTLWLLIQLHESVPGMRYSRYLRLSMAVFGEKLGKILALFPTMYLSGGTCVTLIMIGGGTMKIFFQTICGSNHCHLISLSTIEWYIVFTVSAIVLAQLPNLNSIAGISLIGSISAVTYCTLTWVVSVVKEKPEGVSFEPVENISDLGRVCSILNAIGMIAFAFRGHNLVLEIQGTMPSSLKNPSHLPMWKGVKFSYSIIALCLFPLAIGGYWAYGNLMPNGGILSALDKYHREDTSKVILGITSLLVVVHSLTSFQIYAMPVFDNLEFRYTSNKKKPCPWWIRTGFRVFFGCLAFFISVALPFLPSLAGLIGGIALPVTLAYPCLMWIMINKPKTYTSSWYVNWSLGILGLVLSVLLVFGAIWSIATQGMDVHFFKPQ

NtLHT16

MEERPETELISIPATPRASTPEILTPSGQRSPRGGHTSTGASNKDAKSWTPTSFISPRFLSPIGTPMKRVLVNMKGYLEEVGHLTKLNPQDAWLPITESRNGNAHYAAFHNLNAGIGFQALVLPVAFSFLGWSWGIISLTIAYFWQLYTLWILVQLHEAVPGKRYNRYVELAQAAFGERLGVWLALFPTVYLSAGTATALILVGGETMKLFFQIVCGPLCSSNPLTTVEWYLVFTSLCIVLSQLPNLNSIAGLSLVGAVTAITYATMAWVLSVSQPRPPSISYEPISLPSYTASLFSVLNAMGIIAFTFRGHNLVLEIQATMPSTFKHPAHVPMWKGAKVAYFFIAMCLFPIAIGGFWAYGNLMPSGGMLSALYAFHIHDIPRGLLAMTFLLVVFNCLSSFQIYSMPAFDSFEAGYTSRTNRPCSIWVRSGFRIFFGFVSFFIGVALPFLSSLAGLLGGLTLPVTFAYPCFMWVLIKKPTKYSFNWYFNWILGWLGVAFSLAFSIGGIWSMVNNGLKLRFFKPS

NtLHT17

MGTQAPSDPNYNNDKVDTRTAEEKAIDAWLPITSSRNAKWWYSAFHNVTAMVGAGVLSLPYAMSELGWGPGVTVMVVSWIVTLYTLWQMVEMHEMVPGKRFDRYHELGQHAFGEKLGLWIVVPQQLIVEVGVDIVYMVTGGRSLMKVHELVCKKNEDNIHCTKDIKLSYFIMIFASVHFVLSHLPNFNSISGVSLAAAVMSLSYSTIAWGASVKKGVQSDVDYGYKAHSTSGTVFNFLSGLGEVAFAYAGHNVVLEIQATIPSTPEKPSKIPMWRGVVVAYIVVALCYFPVAFIGYWMFGNSVSDNILVSLEKPTWLIVMANMFVVVHVIGSYQIYAMPVFDMIETVLVKKLRFRPTWYLRFVTRNIYVAFTMFVGITFPFFGGLLGFFGGFAFAPTTYFLPCIMWLAIYKPRRWSLSWITNWICIIFGVLLMVLAPIGGLRSIIVQAKTYKFYN

NtLHT18

MEERPETELISIPATPRASTPEILTPSGQRSPRGGHTSTGASNKDAKSWTPTSFISPRFLSPIGTPMKRVLVNMKGYLEEVGHLTKLNPQDAWLPITESRNGNAHYAAFHNLNAGIGFQALVLPVAFSFLGWSWGIISLTIAYFWQLYTLWILVQLHEAVPGKRYNRYVELAQAAFGERLGVWLALFPTVYLSAGTATALILVGGETMKLFFQIVCGPLCSSNPLTTVEWYLVFTSLCIVLSQLPNLNSIAGLSLVGAVTAITYATMAWVLSVSQPRPPSISYEPISLPSYTASLFSVLNAMGIIAFTFRGHNLVLEIQATMPSTFKHPAHVPMWKGAKVAYFFIAMCLFPIAIGGFWAYGNLMPSGGMLSALYAFHIHDIPRGLLAMTFLLVVFNCLSSFQIYSMPAFDSFEAGYTSRTNRPCSIWVRSGFRIFFGFVSFFIGVALPFLSSLAGLLGGLTLPVTFAYPCFMWVLIKKPTKYSFNWYFNWILGWLGVAFSLAFSIGGIWSMVNNGLKLRFFKPS

NtLHT19

MEERPETELISIPATPRASTPEILTPSGQRSPRGGHTSTGASNKDAKSWTPTSFISPRFLSPIGTPMKRVLVNMKGYLEEVGHLTKLNPQDAWLPITESRNGNAHYAAFHNLNAGIGFQALVLPVAFSFLGWSWGIISLTIAYFWQLYTLWILVQLHEAVPGKRYNRYVELAQAAFGERLGVWLALFPTVYLSAGTATALILVGGETMKLFFQIVCGPLCSSNPLTTVEWYLVFTSLCIVLSQLPNLNSIAGLSLVGAVTAITYATMAWVLSVSQPRPPSISYEPISLPSYTASLFSVLNAMGIIAFTFRGHNLVLEIQATMPSTFKHPAHVPMWKGAKVAYFFIAMCLFPIAIGGFWAYGNLMPSGGMLSALYAFHIHDIPRGLLAMTFLLVVFNCLSSFQIYSMPAFDSFEAGYTSRTNRPCSIWVRSGFRIFFGFVSFFIGVALPFLSSLAGLLGGLTLPVTFAYPCFMWVLIKKPTKYSFNWYFNWILGWLGVAFSLAFSIGGIWSMVNNGLKLRFFKPS

NtLHT20

MVASPDNNAADGRTEEQKAIDEWLPITSSRNAKLWYSAFHNVTAMVGAGILGLPYAMSELGWGPGVTVMVVSWVITLYTLWQMVEMHEMVPGKRFDRYHELGQHVFGNKLGLWIVVPQQLVVEVGLDIVYMVTGGKSFQKIHDLVCKDNCKDIKLTYYIMIFASVHFVLSHLPNFNAISGVSLVAAIMSLSYCTIAWGASIDKGVQPDVEYEYRAKNTGEGIFNFFSGLGEVAFAYAGHNVVLEIQATIPSTAEKPSKGPMWKGVLVAYIIVALCYFPVAIIGYWIFGNSVSDNILISLEKPT

NtLHT21

MTEVEKKKRGSGSEGSSNKDLNDWLPITSSRNAKWYYSAFHNVTAMVGAGVLGLPYAMSQLGWGAGATVMVLSWVITLYTLWQMVEMHEMVPGKRFDRYHELGQHAFGEKLGLWIVVPQQLMVEVGVNIVYMVTGGKSIKKIYDTACPSCRPLKTTYFIMMFSSIHFFLSYCPNFNSITLVSFLAAIMSLSYSTIGWGASVRKGIAPEVDYSPRASTTAGRLFGFLSALGDVAFAFAGHNVVLEIQATMPSSPEKPAKKPMWKGVIFAYIVVALCYFPVAFVGYAVFGKSVEDNILISLEKPAWLIIIANAFVVVHVIGSYQVFAMGVFDMVESYLVKQRKFSPTKTLRFIVRTSYVALTMVLGITFPFFGGLLGFFGGFAFAPTTYFLPCIMWLAIYKPKKFGLSWFTNWICIILGVLLMILAPIGALRQIILQAKDYKFYS

NtLHT22

MQPATMGTQTPNISNYYCSKSADERSAEERAIDAWLPVTSNRNAKWWYSAFHNVTAMVGAGVLGLPYAMAQLGWGPGVAVLVISWIITFYTLWQMVEMHEMVPGKRFDRYHELGQHAFGKKLGLWIIVPQQLIVEVGVDIVYMVTGGQSLQKFYDLVCKKDCKDIKLTYFIMIFASVHFVISHLPDFNSIVGVSLAAAVMSLSYSTIAWGASVKKGVVPDVEYGYKAKSTAGTIFNFFSALGDVAFAYAGHNVVLEIQATIPSTPEKPSKGPMWKGVIVAYIIVAFCYFPVALIGYWMFGNQVKDNILKTLEKPTWLIAMANLFVVIHVIGSYQIYAMPVFDMIETVLVRKLKFKPSWMLRFVTRNIYVAFTMFVGITFPFFNGLLGFFGGFAFAPTTYFLPCIMWLAICKPKKFSLSWIINWICIILGVLLMVIAPIGGLRSIIMQAKGYKFYS

NtLHT23

MVQNNEASMVESGNNSNDGRTNEEINDLNKWLPITASRKAKWWYSAFHNVTAIVGAGVLGLPCAVSQLGWIPGIGMIIISWSVTLYSFWQLVNLHEHVPGKRFDRYPELGKHVFGLKRGYWMVMPQQIIVQVACDIVYMVTGGKSLRESVKMMFHWGRKINQTYYIMFFGVLQLILSQAPNFNSLKVVSFTAAVMSLSYSTIASVASIIKGIEHPKAVNYGLRSHTTAGIIFDIFNSLGTIAFAFAGHSVALEIQATIPSTPERPSKSPMWRGVVVAYAIVAFCYLSVAASGFWAFGNLVADDVLVTLEHPNWLISLANFMVFLHVLGSYQVFAMPVFDTIESFLVKKRHFTPGRPLRLIARSIYVGK
