# Supplementary material for: Comprehensive analysis of the LHT gene family in tobacco and functional characterization of NtLHT22 involvement in amino acids homeostasis
Source: Front Plant Sci. 2022 Sep 13;13:927844. doi: 10.3389/fpls.2022.927844 (PMC9513474; doi:10.3389/fpls.2022.927844)
Supplement: Supplementary Table 2 — The LHT coding sequences of tobacco. [file Table_2.docx]

Table S2 The *LHT* coding sequences of tobacco

*NtLHT1*

ATGGTTTCATCTTCTCCTCCACCAGCTCCAAAGGAAGTTCCTTCAGATGAGAAATGGGCAGAAGATGGTCCTCCTCGCGAAGCCAAATGGTGGTACTCAACTTTTCACACAGTTACTGCAATGGTTGGTGCTGGTGTTCTCAGCCTGCCTTATGCCATGGCCTACTTAGGATGGGGTCCAGGTACGGCGGTTATGATCTTATCATGGTGTATAACCTTACACACAATGTGGCAAATGATACAGCTCCATGAATGTGTGCCTGGAGTTCGTTTCGATCGGTACAAGGATCTTGGAAAACATGCCTTTGGACCAAAACTTGGGGCATGGATAGTACTTCCACAACAGCTAATTGTCCAAGTTGGTTGTGACATTGTGTACATGGTTACTGGAGGAAAGTGTCTGAAGAAGTTCATGGAAATAGCTTGCACTAATTGCACCACAATAAGGCAATCCTATTGGATTTGCATATTTGGTGCAATCCATTTCTTTCTATCACAGCTGCCCAATTTCAATTCTGTTTCTGGTGTTTCATTAGCAGCTGCAGTCATGTCACTGAGCTATTCAACTATAGCATGGGTAGGTTGTATAGGCAAAGGCAGAGTCCCGAACGTGAGCTACGCGTACAAGAAAACAAGTCCAGCTGATTCAATGTTTCGCGTCTTCAACGCGTTAGGTCAAGTTTCCTTTGCTTATGCTGGTCATGCTGTTGTCCTTGAGATACAAGCCACTATTCCATCAACACCTGAGAAGCCCTCCAAAGTTCCAATGTGGAAAGGCGCCGTATGGGCCTATTTTGTCAATGCCCTGTGCTATTTCCCTGTTGCTTTCATCGGGTATTGGGCATTTGGCCAAGACGTCGATGACAATGTGCTCGTGGGACTTGAAAGACCATCTTGGCTTATTGCAGCTGCTAACTTAATGGTGGTTGTTCATGTCATAGGCAGCTATCAGGTTTATGCTATGCCAGTGTTTGATTTGATGGAGCAAAAGATGGTGAAAACCTGGAATTTCCCACCTGGAGTAATGCTGCGCTTCTTCGTTCGTACTGCATATGTTGCTTTCACTTTGTTTCTTGGTGTAACATTCCCTTTCTTTGGGGATCTTCTTGGTTTCTTTGGAGGATTTGGTTTTGCTCCTACTTCTTACTTTCTCCCCTGCATAATGTGGCTTAAGATCAAGAAACCAAGGAGATTCAGCATGTCATGGTTGATAAATTGGGCATGCATATTCATTGGAGTTTTCATTATGATAGCTTCCACAGTTGGTGGATTGAGAAATATTGTTGCTGATTCTTCCACCTATGAGTTCTACTCTTAA

*NtLHT2*

ATGACTAATCTTGATGAAGCTAGCTCTTTACCGAGCTCGTTACCTATAACGCCACGGACGGTAACGGTAGCTCCAACGTTTCATTTTGATGACCAATTTGGCTCTTTACCCATAACTCCACGGACGGCGTCGGTAGCTCAAACGCCGTCCGTAGTATCTTTACCGATAACACCACGGACGGCATCAGTAGCACAAACGCCGTCCATAGTATCGTTACCTCCTTCACAATTTCACTCTCCATCACTTTCTCGATCACCGTTACTTAACGTGGGAGATCATGCCACTAGTGCTGCAAATCGAGCCAGTAAAACCCCAAGGTCACGAGGATTAACGCCGCGTTTCATCACTCCTTTGGGAAGTCCTCTTAGGAAGGCACTTAAAATGACAAGATTAGACCCACAGGATGCTTGGCTACCCATCACTGAGTCACGAAATGGAAACGCATATTACGCTGCGTTTCATACACTTTGTTCTGGGATTGGTATTCAAGCTCTTGTCTTACCTGTTGCCTTTACTATCCTTGGCTGGGCTTGGGGTGTCATTAGCTTAACGGCAGCATTTGTATGGCAGCTCTACACACTCTATTTAATGGTTCAACTTCATGAAAATTATGAAACAGGAATACGTTACAGCAGATACCTGCAACTGGCATGCGCAACATTCGGTGACAAATTAGGCAAACTATGTGCAGCGTTTCCTATCGGATATCTCTCAGCGGGTACATGTTGTGCGCTGATTATAATAGGAGGTTCAACAGCAAAGCTGTTATATCAGACTTTATGCGGAGCAACATGTAGTAATCCCAAGCCATTAACAACGGTGGAATGGTACTTGGTTTTCACTTGTGTTGCAGTGGTTTTAGCACAGTTGCCAAACTTGAATTCTATTGCTGGAATTTCCTTAGTTGGTGCTCTTACGGCCGTTGGATATTGTACTGCATTATGGACGGTTTCAGTTGCTGAGGGTAGACTTCCTAATGTGTCATATGATCCAGTTAGGAAGGGTACTCAAGTTGCTAGGATCTTTGATCTTCTTAATGCTCTTGGTATTATTGCTTTTGCTTTCAGAGGCCACAATCTCATACTTGAAATTCAGGCCACAATGCCTTCAAGTGAGAAGCACCCATCGCGTGTGCCTATGTGGAGGGGTGTGCAATTCTCATATTTACTCATAGCAATGTGCTTATTCCCACTAGCAATTGGTGGCTACTGGGCTTACGGTCATTTGATTCCAGCAAATGGGAGCATGTTAACTGCATTGTTTGCATTCCATAGCCAAGACGTTTCACGATCAGTGTTAGCTCTGATAAGTATTTTTGTGATAATAAACGCGGTGAGTTCCTTTCAAATCTATGGAATGCCAATGTTTGATGATATGGAATCAGCGTACACAACTAGGAGCAAAAAAGCATGTCCGTGGTGGCTCCGTTCGATATTCCGGGCAATTTTCGGATTCGTGTGCTTCTTTATAGCCGTAGCAATTCCATTCTTGGGTAGTTTTGCTGGGCTTATTGGAGGAATTGCACTGCCTGTTACTTTTGCTTATCCATGTTTCATGTGGCTTAAAGTCAAGAAACCCCACAAATATTCTTTAAGTTGGTGGGTAAATTGGGGACTTGGTCTATTGGGAATGGGCTTAAGTGGGATCTTGGTTGCTGCTGGTTTGTATGTTGTCATTGACACTGGTGTTAAAATTAGCTTCTTCAATCCTCAGTGA

*NtLHT3*

ATGCATGACAAAGAAGTTTCAAGGGTTGAGAGTGGGAATAATTCCAACGATGCAACGGCAAACGAACAAATCGATGACGATCTGAATAGATGGTTGCCTATAACAGCTTCCCGAAAAGCCAAATGGTGGTATTCAACATTCCATAATGTAACAGCCGTTGTAGGAGCTGGTGTTCTTGGCTTACCATATGCTGTGTCACAACTCGGCTGGATTCCAGGAATCGGGATGATAATAATATCATGGTTTGTGACATTATACTCACTTTGGCAATTGGTTAATTTGCACGAACATGTCCCGGGGAAGAGATTTGACCGATATCCTGAGTTAGGAAAACATGTATTTGGCCTAAAGAGAGGTTATTGGATAGTAATGCCTCAACAGATGATTGTTCAAGTTGCCAGTGACATAGTTTATATGGTTACAGGGGGGAAATCTCTAAAAGAAAGTATGCATACGATGTTCCATTGGTCTAGAGGGATTAAGCAAACTTACTTTATTTTATTTTTTGGAGTTCTTCAGTTGATACTATCTCAAGCTCCTAATTTTAATTCCTTGAAAGTGGTCTCTTTTACAGCAGCTGTTATGTCTTTGAGTTACTCAACAATTTCGTCAATAGCATCAATTATCAAGGGAGTTGAACATCCCCAACCAGTTAATTATGGTCTACGATCTCATACTCCAGTTGGAATAACATTTGATATTTTCAACAGTTTAGGAACAATTGCATTTGCATTTGCTGGACATAGTGTTGCATTAGAAATTCAAGCAACAATACCTTCAACCCCAGAAAAACCATCTAAAGGGCCAATGTGGCGAGGTGTTACCGTAGCTTATGCAATTGTTGCATTTTGCTATTTAGCTGTTGCTGCCTCTGGATTCTGGGCTTTTGGAAATCTTGTGGATGATGATGTCCTTGTTACACTAAAACATCCACATTGGCTAATTGCTCTTGCAAATTTTATGGTATTTTTGCATGTTCTTGGAAGCTATCAGGTTTTTGCAATGCCTGTTTTTGACATGATTGAGTGTTACTTGGTTAAAAAGCGTCGTTTCACTCCTGGACGACCTCTTCGCCTTATTGCCCGAAGTATTTACGTTGTTGTGACGATGTTCGTTGGAATGTGCTTTCCCTTTTTCGGAGGGTTGCTGGGCTTTTTTGGAGGATTGGCATTTTCATCCACATCATTTTTTCTTTCAATTATTATTGGTGTGAGCATAACCGTCTTGGCGCCAATTGGAGGAGCACGCACCATTATCATCTCAGCAAAGAATTACAAATTCTTCAATTAG

*NtLHT4*

ATGGCTCATGCTAATGAAAAAGAGAAAGATACCAGAACAGAGGAGGAGAAAGCCATAGATGCATGGCTTCCCATCACATCCGATCGAAATGCGAAGTGGTGGTACTCTACTTTTCATAATGTCACTGCCATGGTTGGTGCTGGTGTCCTCAGTCTCCCTTATGCCATGTCTGAGATGGGATGGGGGCCTGGGGTGACAGTAATGCTGCTTTCATGGGCAATAACATTTTATACAATCTGGCAAATGGTAGAGATGCATGAAATGGTACATGGCAAGAGATTTGATAGGTACCACGAGCTAGGTCAACATGCTTTTGGTGAAAAACTAGGACTTTGGATTGTTGTACCCCAACAAATAGTGGTGGACGTGAGTTCTTGCATAATATACATGGTAACAGGTGGCAAGTCGTTGAAGAAATTCCACGAAACAGTTTGCCCTGATTGTCAACCAATAAAGCTGACCTACTTCATCATAATATTCTCCTCAGTCCACTTTGTACTTTCTCATTTGCCAAATTTCAATTCCATATCGTTGGTGTCCTTGGCTGCAGCGGTCATGTCCTTGACATATTCGATTGTTGCTTGGGCAGCGTCTGTAGGGAGAGGGATTGAAGGCAGAGAAGTGAGCTATGAACTTAGGTGTGAAAAGACATCTGATAATATTTTCATGTTCTTGAGTGCACTTGGAGATGTGGCTTTTGCCTATGCTGGCCACAATGTGGTCCTTGAGATTCAAGCCACCATTCCTTCAACACCCGAAAAACCTTCCAAAGGTCCAATGTGGAAAGGTGTATGGGTCGCTTATCTCATCGTCGCCGTCTGTTATCTCCCTGTCGCTTTCATTGGCTATTGGGCCTTTGGTAACGTCGTTGAGGATAACATCTTGCTTTCACTCGAAAAACCTACTTGGGTTATTGCTGCAGCTAACTTGTTTGTTGTCGTTCATGTCATTGGAAGTTACCAGGTTTTCGCAATGCCCGTATTTGACATGATAGAGACATATGCCGTGAAATCAATGAGACTAAAACCATCCACTATTCTTCGGTTCAGTGTGCGTACTACATATGTTGCATTAACATTGTTCGTGGGTCTGACCATACCATTCTTTGGTGGCCTAATGGGATTTTTTGGTGGATTTGCTCTGGCCCCAACATCGTATTACCTTCCTTGCATCATTTGGCTTATCATTGTAAAGCCCAAAAGGTTTGGCTTTTCTTGGTTTATCAATTGGTTCTGCATCATAGTTGGCATACTGCTGACTGTTTTATCACCCATTGGAGGGATGTGGACTCTTATCAAACAAGCTAAGAACTACCGGTTTTACCAGTGA

*NtLHT5*

ATGGTTGAGAGTGGGAATAATTCCGATGATGGAAGGACAAATGAACAAATCGATGATCTGAATAAGTGGTTACCTATAACAGCTTCCCGAAAAGCCAAATGGTGGTATTCAGCATTCCATAATGTAACAGCCATCGTAGGAGCCGGTGTTCTAGGCTTACCATGCGCTGTGTCACAACTCGGCTGGATTCCAGGAATCGGGATGATAATAGTTTCATGGAGTGTGACATTATACTCATTTTGGCAATTGGTTAATTTGCACGAGCATGTCCCGGGGAAGAGATTTGACCGATATCCTGAGTTAGGCAAACATGTTTTTGGCCTAAAGAGGGGTTACTGGATGGTGATGCCTCAACAGATGATTGTTCAGGTTGCCTGTGACATAGTATACATGGTTACAGGGGGGAAGTCTCTAAGGGAAAGTGTGATTATGATGTTCCATTGGGGTCGTCGGATTAATCAAACTTACTATATTATGTTTTTTGGAGTTCTTCAATTGATATTATCCCAAGCTCCTAATTTTAATTCCTTGAAGGTGGTCTCTTTTACAGCTGCTGTTATGTCTTTGAGTTACTCAACAATTGCGTCAATTGCATCGATCATCAAGGGAATTGAGCATCCGAAATCAGCTAATTATGGTCTTCGATCTCATACTACAGCTGGAATAATATTTGATATTTTCAACAGCTTAGGAACCATTGCATTTGCATTTGCTGGACATAGTGTTGCATTAGAAATTCAAGCAACCATCCCTTCAACCCCGGAAAGACCCTCTAAAGGGCCAATGTGGCGGGGTGTTGTCGTGGCTTATGCAATAGTTGCATTTTGCTATTTATCAGTTGCAGCCTCTGGATTCTGGGCTTTTGGCAATCTTGTGGCTGATGACGTCCTTGTTACACTAGAACACCCAAATTGGCTAATTTCTCTTGCAAACTTCATGGTGTTTTTGCATGTTCTTGGAAGCTATCAGGTTTTTGCAATGCCTGTTTTTGACACGATTGAGTCTTTCTTGGTTAAAAAGCGTCATTTCACTCCAGGGCGACCTCTTCGCCTTATTGCCAGAAGCGTTTATGTTGTTCTCACGATGTTCGTTGGAATTTGCATTCCCTTTTTCGGAGGGTTGTTGGGCTTTTTCGGAGGATTGGCATTTTCATCCACATCATTTTTTCTCCCATGCATGATGTGGCTTGTGAGCCACAAACCTAAAAGGTGGAGCTTTCATTGGATCGCTTCTTGGATTTCAATTATTATTGGCGTGAGCATAACCATCTTGGCACCAATAGGAGGAGCACGCACCATTATCATCTCAGCAAAGAATTATACATTATTCTCTCATTCTGAAGAGTGA

*NtLHT6*

ATGTTCGTTGGAATCGCGATCCCTTTCTTTGGTGGGCTTCTTGGATTCTTTGGAGGATTTGCATTTGCCCCAACAACATACTTTCTTCCCTGCATAATGTGGCTTGCAATCTATAAACCAAAGAGATTCAGTCTATCTTGGATTATTAACTGGGCTGATGGAAGAACTGAAGAACAAAAGGCGATAGACGAATGGCTTCCCATTACTTCCTCTAGGAATGCAAAATGGTGGTATTCTGCATTTCACAATGTTACTGCAATGGTTGGAGCTGGTGTCCTCGGTCTTCCTTATGCCATGTCAGAGCTCGGATGGGGACCTGGTGTAACTGTGATGGTGGTATCATGGGTTATAACTTTATACACGCTATGGCAAATGGTTGAGATGCACGAAATGGTTCCAGGGAAACGTTTCGATAGATATCATGAGTTGGGGCAGCATGTTTTTGGTAACAAGCTTGGCCTATGGATCGTGGTGCCTCAGCAATTGGTTGTTGAAGTTGGCCTTGACATTGTTTATATGGTGACCGGAGGAAAATCGTTCCAGAAAATCCATGATTTAGTCTGCAAAGACAATTGCAAAGACATCAAACTGACCTATTACATCATGATATTTGCTTCTGTCCATTTTGTGCTTTCTCATCTTCCCAACTTCAATGCCATATCTGGTGTGTCTTTGGTAGCAGCTATCATGTCCTTAAGTTACTGTACCATTGCTTGGGGGGCTTCAATTGACAAGGGTGTGCAACCAGATGTGGAATATGAATACAGGGCTGAAAACGCAGGGGAAGGTGTTTTCAACTTTTTCAGTGGATTGGGAGAAGTGGCATTTGCATATGCAGGTCATAATGTTGTGTTAGAAATTCAAGCTACAATCCCTTCAACACCTGAGAAACCTTCTAAAGGACCTATGTGGAAAGGAGTCCTTGTTGCTTATATTATTGTGGCTCTATGTTACTTCCCTGTTGCTATTATTGGCTATTGGATATTTGGGAATTCAGTATCAGACAACATTCTTATCTCTTTGGAGAAACCTACTTGGCTCATTGTCTTGGCTAATGCCTTTGTCGTTATCCACATTATTGGGAGCTATCAGTTGTATGCAATTCCGGTGTTTGACATGCTTGAGACTTACCTTATTAAGAAACTTAGGTTCAAGCCAACTTGGTACTTGCGATTTATTACCAGAAACCTTTATGTTGCTTTCACAATGTTTGTTGGAATCATCTTCCCTTTCTTTGGGGGGCTTCTTGGTTTCTTTGGAGGATTTGCTTTTGCCCCAACAACCTATTTTCTCCCTTGCATCATGTGGCTTTCGATCTACAAACCAAAGAGATGGGGTCTCTCTTGGACGACTAACTGGATTTGCATAATAGTGGGAGTAATGTTGACTGTTTTAGCGCCAATTGGTGGCTTAAGAACCATCATTATGCAAGCCAAGGATTACAAATTTTTCTCTTAG

*NtLHT7*

ATGGGAACTCAAGCTCCCTCAGATCCCAACTACAACAATGACAAGGTCGATACAAGAACTGCAGAAGAGAAGGCAATCGATGCGTGGCTCCCTATTACTTCCTCTAGGAATGCGAAATGGTGGTATTCCGCATTTCACAATGTTACTGCTATGGTTGGAGCTGGCGTTCTCAGTCTTCCTTATGCCATGGCAGAGCTTGGATGGGGACCTGGTGTAACAGTGATGGTAGTATCTTGGATTATTACTTTGTACACACTATGGCAAATGGTTGAGATGCACGAAATGGTTCCGGGGAAACGTTTCGATAGATATCATGAATTGGGACAACATGCTTTTGGAGAAAAACTTGGCCTATGGATTGTTGTGCCACAACAATTAATTGTTGAAGTTGGTGTTGACATTGTTTATATGGTAACCGGAGGAAGATCACTAATGAAGGTCCACGAATTGGTTTGCACAAAAAACGAAGACAATATCCATTGCACCAAAGATATTAAACTTTCCTACTTCATCATGATCTTTGCCTCTGTTCATTTTGTGCTCTCCCATCTTCCCAATTTCAATTCCATATCTGGTGTCTCTTTGGCAGCTGCAGTAATGTCCCTAAGTTATTCTACAATTGCTTGGGGGGCATCAGTAAAGAAGGGTGTACAACCGGATGTAGATTACGGGTACAAGGCTCACAGCACGTCAGGAACTGTTTTCAACTTTCTGAGTGGATTGGGAGAAGTGGCTTTTGCATATGCGGGTCATAACGTGGTTTTGGAGATTCAAGCTACGATCCCTTCAACACCTGAGAAACCTTCGAAAATACCTATGTGGAGAGGAGTTGTTGTTGCTTACATAGTTGTGGCTCTCTGTTACTTCCCTGTTGCTTTTATTGGCTATTGGATGTTTGGGAATTCTGTAGAAGACAACATTCTCGTGTCTTTGAACAAACCTACATGGCTAATTGCCATGGCTAACATGTTTGTCGTCGTTCACGTTATTGGGAGCTATCAGATCTATGCAATGCCAGTGTTTGACATGCTCGAGACTGTGCTTGTTAAGAAACTTAGGTTCAGCCCTACTTGGTACCTGCGATTTGTTACCAGAAACATTTATGTTGCTTTCACAATGTTTGTTGGAATCACCTTCCCTTTCTTCGGGGGGCTTCTTGGATTTTTTGGAGGATTTGCTTTTGCACCAACAACATATTTTCTCCCTTGCATTATGTGGCTTGCAATCTACAAGCCAAGGAGATGGAGTCTCTCTTGGATTGCTAATTGGATTTGCATAATTTTTGGAGTTTTATTGATGGTTTTAGCACCAATTGGTGGTTTGAGATCCATCATAGTACAAGCCAAGACCTACAAATTTTACAATTAG

*NtLHT8*

ATGGGAAGTGAATTGGTGGAAATTAAGATGAGCCAATCACCACTAAAAATTGGTGATAAAGAAGTGCAAAATACAGTTTCTTTAACACCATCTCCAATTCTTGATTCTATACCAAAAACACCCAAAAGTCCATTTGGTGCAAGAATAATGACACCATTGGCTAGTCCTATGAAAAAAGCTTTAACATATATGGAAGAAATTGGTCATTTCACTAAACTTGATCCTCAAGATGCTTGGCTTCCTATTACTGAGTCAAGAAATGGGAATGCATATTATGCTGCATTTCATACACTTAGTTCTGGAATTGGAGTCCAAGCTCTTGTCCTTCCTCTTGCTTTTATTACACTTGGATGGATATGGGGGATAATAAGCCTCTCAATAATATTTATGTGGCAATTATACACACTCTGGTTACTCATCCAACTTCATGAATCTGTCCCTGGCATGCGTTATAGTCGATATCTTCGCCTCTCAATGGCTGCCTTCGGTGAAAAGCTGGGGAAAATTTTAGCACTATTTCCAACCATGTACCTATCAGGTGGTACTTGCGTTACACTTATTATGATCGGAGGTGGAACTATGAAGATTTTCTTCCAAACTATCTGTGGATCTAATCATTGCCATTTAACCTCTCTAAGTACAATAGAGTGGTACATTGTATTCACTGTTTCAGCCATAATTCTTGCTCAGCTTCCTAATTTAAATTCCATTGCTGGAATTTCTCTCGTCGGCTCGATCTCGGCAGTGACTTATTGTACCTTGACATGGGTAGTTTCTGTTGTCAAAGAAAGGCCAGAAGATGTTTCTTTTGAAACTGTTGAAAATAAATCTGATTTGGAAAGAGTTTGTAGCATTTTGAATGCTATTGGAATGATAGCTTTTGCTTTCAGGGGACATAATCTTGTCCTTGAGATACAGGGTACAATGCCTTCTAGCTTAAAGAACCCATCTCATGTGCCCATGTGGAAAGGAGTCAAGTTCTCATATTCTATTATTGCTTTGTGTTTGTTTCCACTGGCAATTGGAGGCTACTGGGCTTATGGAAACCTGATGCCAAATGGAGGGATATTGAGTGCATTGGACAAATACCATGGAAAAGACACATCAAAAGTAATTTTAGGAATAACAAGTTTACTGGTGGTTGTCCATAGCCTTACATCATTCCAAATCTATGCAATGCCAGTTTTTGATAATTTGGAGTTTAGGTACACCAGCAACAAGAAAAAACCCTGTCCATGGTGGCTTAGAACAGGGTTTAGAGTATTCTTTGGATGCCTAGCATTTTTCATATCAGTGGCACTTCCCTTTTTGCCTAGTTTGGCTGGTCTAATTGGGGGAATTGCTTTGCCAGTTACTTTGGCATATCCTTGTCTAATGTGGATAATGATCAAGAAACCTCAGACATATACTTCAACTTGGTTTGTTAATTGGTCTCTTGGACTTTTAGGCTTGGTTCTAAGTGTACTTTTGGTTTTTGGTGCTATATGGACTATAGCAATTCAAGGTATGGATGTCCACTTTTTCAAGCCACAGTAA

*NtLHT9*

ATGGGAGATATAGAAAGGGTGTCATCATCTTTCTCTTCATTGAAAATCATACCCATTGATAATGACGATCGCTTTGACAATAATCAGTCGGAAGGAAGAGATTCGCCGTCATGCATGGCGGCGGTCGACGGCGGCATGGAGAAGAAGAATATGAATATTCCAGAAGAAGAAGTAGAAAGTTACTTGCCTATAACGGAATCAAGAAAGGGGAATGCTTATACGGCGGCGTTTCATTTATTGTGTTCTGGCATTGGAACTCCAGCTCTTGTTCTCCCCTTTGCTTTCACGTCTTTGGGATGGTCGTGGGGAATAATAATTTTGACGGTGGTTTTTGCATGGCGGCTGTATACTATGTGGCTATTAGTTCATCTTCACGAATCAAACTCTGGAACTCGTTATAGCAGATACCTTCAACTCTCTATTGCAGCTTTCGGACTAAAATTGGGAAAATGTCTAGCAATATTCCCAATAATGTACCTATCAGGGGGCACTTGTGTTATGTCCATTATAGCAGGGGGAGGTACCCTTCAACTTTTCTACAATGCAATATGTGGAAATGATCACAATTGCCACCACAGATCTTTGAGTGGAGTTCAGTGGTTCTTGTTGTTTATATGCCTTGCCATTTTAATTGCCCAATTTTGCCCCAATTTGCACTCCTTATCTTGGGTTTCCTTTGTTGGCTCAGTCATGGGCGTGGCCTATTTAACTCTAATATGGGCACTCTCCATCAGTAAAGGTAGACCCAATGGAGTCTCCTACAACCCATCTGATAATGCAACAACAACAATGGCTCGATTTCGTGCTATCCTAAATGGTGTTGCTATAATTGTCATTGCTTTCAGAGGTCACAATGTTGTTTTGGAAATACAGGGGACATTACCTACCAATCCTAAACATCCTACACGGACTAGTATGTGGAGAGGAGTAGTGTCATCATACTCTTTTATCGCAATGTGTATATTTCCCCTGGCAATTGGAGGATATTGGTCTTATGGAAATCTGATGCCTGCAAGTGGAATTATGACCGCAATTGCAAAATACCACCAAGAGAGCACACCAAAGTGGTTAACAGGCACAATATACATAATGGTGATAATCCAATCTTTATGCACATTTCAAATATATGCAATGCCCGTATTTGACAACTTTGAAAGAATATATGTAAGCAAGCAACACAAGGCGTGCCCGAGGTGGGTAAAATTATGCATCAAACTCTTCTTTGGCGGATTGACATATTTCATATCAGTGGCATTCCCATTTTTGGGAAGTTTGGCTGCTTTTGTGGGTGGAATTGCACTGCCTTTATCTCTGGTTTACCCTTGCTTCATGTGGATTTCAATCAAGAAACCTAGCAGAAACAGCTTAATGTATTGCCTCAACATGATTCTTGGATGTTTGGGTATGTTGATTAGCATTGTACAAGTTGCTGGTGCGTTGTGGAATCTGGTGGTTGAAAAATTTGACGCCAATTTTTTTAGTCCTTAG

*NtLHT10*

ATGGTTGGAGCTGGTGTCCTCGGTCTTCCTTATGCCATGTCAGAGCTCGGATGGGGACCTGGTGTAACTGTGATGGTGGTATCATGGGTTATAACTTTATACACGCTATGGCAAATGGTTGAGATGCACGAAATGGTTCCAGGGAAACGTTTCGATAGATATCATGAGTTGGGGCAGCATGTTTTTGGTAACAAGCTTGGCCTATGGATCGTGGTGCCTCAGCAATTGGTTGTTGAAGTTGGCCTTGACATTGTTTATATGGTGACCGGAGGAAAATCGTTCCAGAAAATCCATGATTTAGTCTGCAAAGACAATTGCAAAGACATCAAACTGACCTATTACATCATGATATTTGCTTCTGTCCATTTTGTGCTTTCTCATCTTCCCAACTTCAATGCCATATCTGGTGTGTCTTTGGTAGCAGCTATCATGTCCTTAAGTTACTGTACCATTGCTTGGGGGGCTTCAATTGACAAGGGTGTGCAACCAGATGTGGAATATGAATACAGGGCTGAAAACGCAGGGGAAGGTGTTTTCAACTTTTTCAGTGGATTGGGAGAAGTGGCATTTGCATATGCAGGTCATAATGTTGTGTTAGAAATTCAAGCTACAATCCCTTCAACACCTGAGAAACCTTCTAAAGGACCTATGTGGAAAGGAGTCCTTGTTGCTTATATTATTGTGGCTCTATGTTACTTCCCTGTTGCTATTATTGGCTATTGGATATTTGGGAATTCAGTATCAGACAACATTCTTATCTCTTTGGAGAAACCTACTTGGCTCATTGTCTTGGCTAATGCCTTTGTCGTTATCCACATTATTGGGAGCTATCAGTTGTATGCAATTCCGGTGTTTGACATGCTTGAGACTTACCTTATTAAGAAACTTAGGTTCAAGCCAACTTGGTACTTGCGATTTATTACCAGAAACCTTTATGTTGCTTTCACAATGTTTGTTGGAATCATCTTCCCTTTCTTTGGGGGGCTTCTTGGTTTCTTTGGAGGATTTGCTTTTGCCCCAACAACCTATTTTCTCCCTTGCATCATGTGGCTTTCGATCTACAAACCAAAGAGATGGGGTCTCTCTTGGACGACTAACTGGATTTGCATAATAGTGGGAGTAATGTTGACTGTTTTAGCGCCAATTGGTGGCTTAAGAACCATCATTATGCAAGCCAAGGATTACAAATTTTTCTCTTAG

*NtLHT11*

ATGGCTCATGCTAATGAAAAAGAGATAGATACCAGAACGGAAGAGGAGAAAGCCATAGATGCATGGCTTCCCATCACATCCGATCGAAATGCGAAGTGGTGGTACTCTACTTTTCATAATGTCACTGCCATGGTTGGTGCTGGTGTCCTCAGTCTCCCTTATGCCATGTCTGAGATGGGATGGGGGCCTGGGATAACAGTAATGCTTCTTTCATGGGCTATAACATTTTATACAATCTGGCAAATGGTAGAGATGCATGAAATGGTACCTGGGAAGAGATTTGATAGGTACCACGAGCTAGGTCAACATGCATTTGGTGAAAAACTAGGACTTTGGATTGTTGTACCCCAACAAATAGTGGTGGACGTGAGTTCCTGCATAATATACATGGTAACAGGTGGAAAGTCGTTGAAGAAATTCCACGAAACAGTTTGCCCTGATTGTCAACCAATAAAGCTGACCTACTTCATCATAATATTCTCCTCAGTCCACTTTGTACTATCTCACTTGCCAAATTTCAATTCCATATCGTTGGTGTCCTTGGCTGCAGCGGTCATGTCGTTGACATATTCGATTGTTGCTTGGGCATGCTCTATAGGGAGAGGGATTGAAGGCAGAGAAGTGAGCTATGAACTTAGGGGTGAAAAGACATCTGATAATATTTTCATGTTCTTGAGTGCACTTGGAGATGTGGCTTTTGCCTATGCTGGCCACAATGTGGTCCTTGAGATTCAAGCCACCATTCCTTCAACACCCGAAAAACCTTCCAAAGGTCCAATGTGGAAAGGTGTATGGGTCGCTTATCTCATCGTCGCCGTCTGTTATCTCCCTGTCGCTTTCATTGGCTATTGGGCCTTTGGTAATGTCGTCGAGGATAACATCTTGCTTTCACTCGAAAAACCTGTTTGGGTTGTTGCTGCTGCTAACTTGTTTGTTGTCGTTCATGTCATTGGAAGTTACCAGGTTTTCGCAATGCCCGTGTTTGACATGATAGAGACATATGCCGTGAAATCAATGAGACTAAAACCATCCACTCTCCTTCGGTTCGGTGTGCGTACTACATTTGTTGCATTTACATTGTTCGTGGGTATGACCATACCATTCTTTGGTGGCCTAATGGGATTTTTTGGTGGATTTGCTCTGGCCCCAACATCGTATTACCTTCCTTGCATCATTTGGCTTATCATTGTAAAGCCCAAAAGGTTTGGCTTTTCTTGGTTTATGAATTGGTTCTGCATCATAGCTGGCATACTGCTGACTGTTTTATCACCCATTGGAGGGATGTGGACTCTTATCAAACAAGCCAAGAACTACCGGTTTTACCAGTGA

*NtLHT12*

ATGGTTTCATCTTCTCCTCCACCAGCTCCAAAGGAAGTTCCTTCAGATGAGAAATGGGCAGAAGATGGTCCTCCTCGCGAAGCGAAATGGTGGTACTCAACTTTTCACACAGTTACTGCGATGGTTGGTGCTGGTGTTCTCAGCTTGCCTTATGCCATGGCCTACTTAGGATGGGGTCCAGGGACGGTAGTTATGATCTTATCATGGTGTATAACCTTACACACAATGTGGCAAATGATACAACTCCATGAATGTGTTCCCGGAGTTCGTTTCGATCGGTACAAGGATCTTGGTAAACATGCCTTTGGACCAAAACTTGGGGCATGGATAGTACTTCCACAACAACTAATTGTCCAAGTTGGTTGTGACATTGTGTACATGGTTACTGGAGGAAAGTGTCTGAAGAAGTTCATGGAAATAGCTTGCACAAATTGCACCACAATAAGGCAATCCTATTGGATTTGCATATTTGGTGCAATCCATTTCTTTCTATCACAGCTTCCCAATTTCAATTCTGTTTCTGGTGTTTCATTAGCAGCTGCAGTCATGTCACTGAGCTATTCAACTATAGCATGGGTAGGTTGTGTAGGCAAAGGCAGAGTCCCGAACGTGAGCTACGCGTACAAGAAAACAAGTCCAGCTGATTCTATGTTTCGCGTCTTTAACGCGTTAGGTCAAGTTTCCTTTGCTTATGCTGGTCATGCTGTTGTCCTTGAGATACAAGCCACTATTCCATCGACACCTGAGAAGCCCTCGAAAGTTCCAATGTGGAAAGGCGCCGTATGGGCCTATTTTGTCAATGCCCTGTGCTATTTCCCCGTTGCTTTCATCGGGTATTGGGCATTTGGCCAAGATGTCGATGACAACGTGCTCGTGGGACTTGAAAGGCCATCTTGGCTTATTGCAGCTGCTAACTTAATGGTGGTTGTTCATGTCATAGGCAGCTATCAGGTTTATGCTATGCCAGTGTTTGATTTGATGGAGCAAAAAATGGTGAAAACCTGGAATTTCCCACCTGGAATAATGCTGCGCTTCTTCGTTCGTACTGCATATGTTGCTTTCACTTTGTTTCTTGGTGTAACATTCCCTTTCTTTGGTGATCTTCTTGGTTTCTTTGGAGGATTTGGTTTTGCTCCTACTTCTTATTTTCTCCCCTGCATAATGTGGCTTAAGATCAAGAAACCAAGGAGATTCAGCATGTCATGGTTGATAAATTGGGCATGCATATTCATTGGAGTTTTCATTATGATAGCTTCCACAATTGGTGGATTGAGAAATATTGTTGCTGATTCTTCCACCTATGAGTTCTACTCTTGA

*NtLHT13*

ATGGAAAAAGGAGGAGATAATGAAGATAAGAGGAGGGAGGAGGAGAAAGCAATAGATGAATGGCTACCGATAACCTCGGACCGGAATGCAAAATGGTGGTATTCAACCATGCACAATGTTACTGCCATGGTTGGTGCTGGTGTTCTTAGTCTACCTTATGCCATGTCTCAGATGGGATGGGGAGCTGGTGTAACGGTATTGTTACTGTCATGGATTATAACATTCTACACAATCTGGCAAATGGTGGAAATGCATGAAATGATACCAGGCAAGAGATTTGACAGGTACCATGAGCTTGGCCAATATGCTTTTGGTGAAAAACTTGGTCTCTGGATTGTTGTACCCCAACAAATTGTAGTTGAAGTTAGCACTTGTATTATTTACATGGTCACTGGTGGCAAATCCTTGAAAAAATTCCAAGAAATCCTTTTCCCAAATGCCAAACCTATCAAACTCACTTATTTCATCTTGATTTTCTCCTCTTTCCAATTTATCCTCTCTCACTTGCCAAATTTCAACTCCATCTCCTCCGTCTCCTTCGTCGCGGCGATTTTGTCCATGACTTACTCAGCTATAGCATGGACTGTTTCACTAAAGGAAAGTGGGAGAGAAGTTAGTTATGGTCCAACAAGTGAAAAAACATCAGATAATGTGTTTATGTTTCTGAGTGCATTAGGAAATGTAGCATTTGCATATGCTGGACATAATGTAGTACTTGAAATTCAAGCAACAATTCCTTCGACAGAAGATGCACCATCCAAAAAAGCAATGTGGAAAGGTGTATTAACAGCTTATATTATAGTGGCCTTGTGTTATTTGCCTGTGGCTTTCATTGGATATTGGGGTTTTGGTAATGGAGTTGATGATAACATCTTGCTCACACTACATACACCTACATGGCTTATTGCAACTGCTAACATTTTTGTTGTCGCTCATGTCATTGGGAGTTACCAGGTTTATGCAATGCCAGTGTTTGATATGATAGAGACATACGCGGTGAAGTCATTGAGATATAAACCTTCCACTATTCTACGTGTTTGTGTGCGCACGGTTTTTGTCGCATTTACATTGGTTGTGGGCATGACAATACCATTCTTTGGTGGTTTGATGGGATTCTTTGGAGGTTTTGCTCTGGCACCAACCTCATATTATCTTCCATGCATCATCTGGCTTATTATAAAAAAGCCCAGACGGTTTGGCTTGTCATGGTGTACGAACTGGCTGTGCATCATAGTGGGTGTACTTTTGACTCTTACATCTCCCATTGGTGGATTATGGAGTATCATCAAATCTGCCAAGACTTATCGCTTCTACACCTGA

*NtLHT14*

ATGACTGGAGAGGAGAAGAAAAAGAGAGGATCAGGATCTGAGGGAAGCAGTAATAAGGATCTGAACGATTGGCTACCAATCACATCATCAAGGAATGCAAAGTGGTATTATTCGGCGTTTCACAATGTCACTGCCATGGTTGGTGCTGGTGTTCTTGGCCTCCCTTATGCCATGTCTCAGTTGGGTTGGGGTGCTGGAGCAACAGTAATGGTATTATCATGGGTGATAACACTATACACCTTATGGCAAATGGTAGAGATGCATGAGATGGTTCCAGGTAAAAGATTTGACAGATACCATGAGCTGGGGCAACATGCATTTGGTGAAAAACTTGGTCTTTGGATTGTGGTTCCTCAGCAGCTAATGGTTGAAGTTGGTGTGAACATAGTGTATATGGTCACTGGTGGCAAATCCATCAAAAAGATATATGATACAGCTTGTCCTAGTTGTAGACCATTGAAAACCACCTATTTTATCATGATGTTTAGTTCTATTCACTTCTTCCTCTCCCATTGTCCCAATTTCAACTCCATCACTCTTGTCTCCTTCCTTGCTGCCATCATGTCTCTCAGTTATTCAACCATAGGTTGGGGAGCATCAGTACATAAAGGAATATCACCAGAGGTTGATTACAGTCCAAGGGCATCAACAACTACAGGAAGAGTATTTGGTTTCTTGAGTGCTTTAGGAGATGTTGCTTTTGCATTTGCTGGTCATAATGTTGTCTTGGAAATTCAGGCAACTATGCCTTCTTCTCCTGAAAAACCAGCCAAGAAACCTATGTGGAAAGGAGTCATTTTTGCCTACATTGTTGTGGCTTTGTGTTACTTTCCTGTGGCTTTTGCTGGCTATGCAGTTTTTGGGAAAAGTGTTGAGGATAATGTCTTGATCTCCCTTGAGAAACCTGCTTGGCTTATTATCATTGCTAACGCCTTCGTTGTTGTCCATGTTATTGGAAGCTATCAGGTGTTTGCAATGGGTGTGTTTGACATGGTGGAATCTTACTTGGTGAAGCAAAGGAAATTCACTCCAACTAAAACGCTACGGTTTATTGTTCGGACTAGTTATGTTGCCCTAACAATGTTTCTTGGTATAACATTCCCATTCTTTGGTGGGCTACTGGGTTTCTTTGGAGGATTTGCATTTGCTCCGACCACTTACTTCCTTCCTTGTATCATGTGGCTTGCAATCTACAAACCTAAAAAGTTTGGCTTGTCTTGGTTCACTAATTGGATATGCATCATACTTGGTGTTCTTCTGATGATTTTAGCTCCCATTGGTGCCTTGAGGCAAATCATATTGCAAGCCAAGGACTACAAGTTCTATTCTTGA

*NtLHT15*

ATGACTGGAGAGGAGAAGAAAAAGAGAGGATCAGGATCTGAGGGAAGCAGTAATAAGGATCTGAACGATTGGCTACCAATCACATCATCAAGGAATGCAAAGTGGTATTATTCGGCGTTTCACAATGTCACTGCCATGGTTGGTGCTGGTGTTCTTGGCCTCCCTTATGCCATGTCTCAGTTGGGTTGGGGTGCTGGAGCAACAGTAATGGTATTATCATGGGTGATAACACTATACACCTTATGGCAAATGGTAGAGATGCATGAGATGGTTCCAGGTAAAAGATTTGACAGATACCATGAGCTGGGGCAACATGCATTTGGTGAAAAACTTGGTCTTTGGATTGTGGTTCCTCAGCAGCTAATGGTTGAAGTTGGTGTGAACATAGTGTATATGGTCACTGGTGGCAAATCCATCAAAAAGATATATGATACAGCTTGTCCTAGTTGTAGACCATTGAAAACCACCTATTTTATCATGATGTTTAGTTCTATTCACTTCTTCCTCTCCCATTGTCCCAATTTCAACTCCATCACTCTTGTCTCCTTCCTTGCTGCCATCATGTCTCTCAGTTATTCAACCATAGGTTGGGGAGCATCAGTACATAAAGGAATATCACCAGAGGTTGATTACAGTCCAAGGGCATCAACAACTACAGGAAGAGTATTTGGTTTCTTGAGTGCTTTAGGAGATGTTGCTTTTGCATTTGCTGGTCATAATGTTGTCTTGGAAATTCAGGCAACTATGCCTTCTTCTCCTGAAAAACCAGCCAAGAAACCTATGTGGAAAGGAGTCATTTTTGCCTACATTGTTGTGGCTTTGTGTTACTTTCCTGTGGCTTTTGCTGGCTATGCAGTTTTTGGGAAAAGTGTTGAGGATAATGTCTTGATCTCCCTTGAGAAACCTGCTTGGCTTATTATCATTGCTAACGCCTTCGTTGTTGTCCATGTTATTGGAAGCTATCAGGTGTTTGCAATGGGTGTGTTTGACATGGTGGAATCTTACTTGGTGAAGCAAAGGAAATTCACTCCAACTAAAACGCTACGGTTTATTGTTCGGACTAGTTATGTTGCCCTAACAATGTTTCTTGGTATAACATTCCCATTCTTTGGTGGGCTACTGGGTTTCTTTGGAGGATTTGCATTTGCTCCGACCACTTACTTCCTTCCTTGTATCATGTGGCTTGCAATCTACAAACCTAAAAAGTTTGGCTTGTCTTGGTTCACTAATTGGATATGCATCATACTTGGTGTTCTTCTGATGATTTTAGCTCCCATTGGTGCCTTGAGGCAAATCATATTGCAAGCCAAGGACTACAAGTTCTATTCTTGA

*NtLHT16*

ATGAGCCAATCAACACTAAAAATTGGTAGTGAAGAAGTGCAAAATACAGTTTCTTTGACACCATCTCCAAATCTTGATTCTATACCAAAAACACCCAAAAGTCCATTTGGTACAAGAATAATGACACCATTAGCTAGCCCCATGAAAAAAGCCTTAACATATATGGAAGAAATTGGTCATTTCACTAAGCTTGATCCTCAAGATGCTTGGCTTCCTATTACTGAGTCAAGAAATGGGAATGCATATTATGCTGCATTTCATACACTTAGTTCTGGAATTGGAGTTCAAGCTCTTGTCCTTCCTCTTGCTTTTATTACACTTGGATGGATATGGGGAATAATAAGCCTCTCAATAATATTTATGTGGCAATTATACACACTCTGGTTACTCATCCAACTTCATGAATCTGTCCCTGGCATGCGTTATAGTCGATATCTTCGCCTCTCAATGGCTGTCTTCGGTGAAAAGCTGGGGAAAATTTTAGCACTATTTCCAACCATGTACCTATCAGGTGGTACTTGCGTTACACTTATTATGATCGGAGGTGGAACTATGAAGATTTTCTTCCAAACTATTTGTGGTTCTAATCATTGCCATTTAATCTCTCTAAGTACAATAGAGTGGTACATTGTATTCACTGTTTCAGCCATAGTTCTTGCTCAGCTTCCTAATTTAAATTCCATTGCTGGAATTTCTCTAATCGGTTCAATCTCAGCAGTGACTTATTGTACCTTGACATGGGTAGTTTCTGTTGTCAAAGAAAAGCCAGAAGGGGTTTCTTTTGAACCTGTTGAAAATATATCTGATTTGGGAAGAGTTTGTAGCATTTTGAATGCTATTGGAATGATAGCTTTTGCTTTCAGGGGACATAATCTTGTCCTTGAGATTCAGGGTACAATGCCTTCTAGCTTAAAGAACCCATCTCATTTGCCCATGTGGAAAGGAGTCAAGTTCTCATATTCTATTATTGCTTTGTGTTTGTTTCCACTGGCAATTGGAGGCTACTGGGCTTATGGAAACCTGATGCCAAATGGAGGGATATTGAGTGCATTGGACAAGTATCACAGAGAAGACACATCAAAAGTAATTCTAGGAATAACAAGTTTACTGGTGGTAGTCCATAGCCTTACATCATTCCAAATTTATGCAATGCCAGTTTTTGATAATTTGGAGTTTAGGTACACAAGCAACAAGAAGAAACCCTGTCCATGGTGGATTAGAACAGGGTTTAGGGTATTTTTTGGATGTCTAGCATTTTTCATATCAGTGGCACTCCCCTTTTTGCCTAGTTTGGCTGGTCTAATTGGGGGAATTGCTCTGCCAGTTACCTTGGCATATCCTTGTCTGATGTGGATAATGATCAACAAACCTAAGACATACACTTCAAGTTGGTATGTTAATTGGTCTCTTGGAATTTTAGGCTTGGTTCTAAGTGTTCTTTTGGTATTTGGTGCTATCTGGTCTATAGCAACTCAGGGTATGGATGTTCACTTCTTCAAGCCACAATGA

*NtLHT17*

ATGGAGGAAAGACCTGAGACTGAACTCATTTCAATACCAGCAACTCCACGTGCTTCAACGCCTGAGATTCTAACGCCGTCAGGTCAAAGGTCACCTAGGGGAGGACATACATCAACTGGAGCTTCTAATAAAGATGCTAAATCTTGGACACCAACTTCATTTATTTCGCCTAGATTCTTGAGCCCTATTGGTACTCCAATGAAAAGGGTGTTGGTTAATATGAAAGGTTATTTGGAGGAAGTTGGGCATTTGACTAAGCTTAATCCTCAAGATGCTTGGCTTCCTATTACTGAATCTCGTAATGGGAATGCTCACTATGCTGCTTTTCATAATCTCAATGCTGGTATTGGGTTTCAAGCTTTGGTCTTGCCTGTTGCTTTCTCATTTCTTGGATGGAGTTGGGGAATAATTTCCTTAACCATAGCTTATTTCTGGCAACTTTATACTTTATGGATCTTGGTTCAGCTGCATGAAGCAGTTCCTGGGAAGAGATACAATAGATACGTGGAACTAGCGCAAGCTGCATTTGGTGAAAGACTGGGTGTTTGGCTTGCCCTCTTCCCTACTGTTTACCTATCTGCAGGGACTGCGACAGCTTTGATTCTCGTGGGAGGTGAAACCATGAAGTTGTTCTTTCAAATTGTTTGCGGTCCACTCTGTTCATCAAATCCTTTAACAACTGTAGAGTGGTATCTGGTTTTCACTTCCCTCTGCATTGTTCTATCCCAACTCCCGAACTTGAACTCCATTGCTGGACTCTCCCTCGTTGGAGCAGTGACAGCCATCACATACGCCACTATGGCATGGGTCCTCTCTGTAAGCCAACCAAGACCACCTTCGATTTCATATGAACCCATTTCATTGCCTTCCTACACAGCTTCTCTCTTTTCTGTCCTGAACGCCATGGGTATCATAGCATTTACTTTTAGAGGGCACAATTTAGTTCTAGAAATTCAGGCAACAATGCCATCAACCTTCAAGCACCCTGCTCATGTGCCAATGTGGAAAGGAGCAAAAGTTGCATATTTCTTCATAGCCATGTGCCTGTTCCCTATTGCAATTGGAGGTTTCTGGGCTTATGGAAACCTTATGCCATCGGGAGGAATGCTAAGTGCTCTATACGCCTTTCACATTCATGATATTCCAAGAGGACTTCTCGCCATGACGTTTCTCTTAGTCGTGTTCAACTGTTTAAGTAGTTTCCAGATATACTCAATGCCAGCATTCGATAGTTTTGAAGCAGGCTACACCAGCCGTACCAACCGACCATGCTCAATCTGGGTCCGTTCTGGATTCAGAATATTTTTCGGATTCGTTTCGTTCTTTATTGGAGTGGCGCTTCCGTTCCTGTCAAGTCTTGCAGGGTTGTTAGGAGGACTTACACTTCCGGTAACATTTGCTTATCCTTGCTTCATGTGGGTTCTAATAAAGAAGCCTACAAAGTATAGCTTTAACTGGTATTTCAACTGGATCCTTGGATGGTTAGGAGTTGCTTTTAGCTTGGCTTTTTCCATTGGAGGTATTTGGAGTATGGTCAATAATGGACTTAAACTCAGGTTCTTTAAGCCCAGCTAA

*NtLHT18*

ATGGGAACTCAAGCTCCTTCAGATCCCAACTACAACAATGACAAGGTTGATACAAGAACTGCAGAAGAGAAGGCAATCGATGCGTGGCTTCCCATTACTTCTTCTAGGAATGCAAAATGGTGGTATTCAGCATTTCACAATGTTACTGCTATGGTTGGTGCTGGTGTTCTCAGTCTTCCTTATGCCATGTCAGAGCTTGGATGGGGACCCGGTGTCACAGTGATGGTAGTATCTTGGATTGTTACTTTGTATACACTATGGCAAATGGTTGAAATGCATGAAATGGTTCCAGGGAAACGTTTCGATAGATATCATGAATTAGGGCAACATGCTTTTGGAGAAAAACTTGGCCTATGGATTGTTGTGCCACAGCAGTTAATTGTTGAAGTTGGTGTTGACATTGTTTATATGGTAACTGGAGGAAGATCACTAATGAAGGTCCACGAATTGGTTTGCAAAAAAAACGAAGACAATATCCATTGCACCAAAGATATTAAACTTTCCTACTTCATTATGATCTTTGCCTCTGTTCATTTTGTGCTTTCCCATCTTCCCAATTTCAATTCCATATCTGGTGTCTCTTTGGCTGCCGCAGTAATGTCCCTAAGTTATTCTACAATCGCTTGGGGGGCATCAGTAAAGAAGGGTGTACAATCGGATGTAGATTACGGGTACAAGGCTCACAGCACGTCAGGAACTGTTTTCAACTTTCTGAGTGGATTGGGAGAAGTGGCTTTTGCATATGCGGGTCATAACGTGGTTTTGGAGATTCAAGCAACAATCCCTTCAACACCTGAAAAACCTTCGAAAATACCTATGTGGAGAGGAGTTGTTGTTGCTTACATAGTTGTGGCTCTGTGTTACTTCCCTGTCGCATTTATTGGCTATTGGATGTTTGGGAATTCAGTATCAGACAATATTCTCGTGTCTTTGGAGAAACCTACTTGGCTCATTGTCATGGCTAACATGTTTGTCGTCGTCCATGTTATTGGGAGCTATCAGATCTACGCAATGCCAGTGTTTGACATGATCGAGACTGTGCTTGTTAAGAAACTTAGGTTCAGGCCTACTTGGTACTTGAGATTTGTTACCAGAAATATTTATGTTGCTTTCACAATGTTTGTTGGAATCACCTTCCCTTTCTTTGGGGGACTTCTTGGATTCTTTGGAGGATTTGCTTTTGCTCCAACAACTTATTTTCTTCCTTGCATCATGTGGCTTGCAATCTACAAGCCAAGGAGATGGAGTCTCTCTTGGATTACTAATTGGATTTGCATAATTTTTGGAGTTTTATTGATGGTTTTAGCACCAATTGGTGGTTTGAGATCTATCATAGTACAAGCCAAGACCTACAAATTTTACAATTAG

*NtLHT19*

ATGGAGGAAAGACCTGAGACTGAACTCATTTCAATACCAGCAACTCCACGTGCTTCAACGCCTGAGATTCTAACGCCGTCAGGTCAAAGGTCACCTAGGGGAGGACATACATCAACTGGAGCTTCTAATAAAGATGCTAAATCTTGGACACCAACTTCATTTATTTCGCCTAGATTCTTGAGCCCTATTGGTACTCCAATGAAAAGGGTGTTGGTTAATATGAAAGGTTATTTGGAGGAAGTTGGGCATTTGACTAAGCTTAATCCTCAAGATGCTTGGCTTCCTATTACTGAATCTCGTAATGGGAATGCTCACTATGCTGCTTTTCATAATCTCAATGCTGGTATTGGGTTTCAAGCTTTGGTCTTGCCTGTTGCTTTCTCATTTCTTGGATGGAGTTGGGGAATAATTTCCTTAACCATAGCTTATTTCTGGCAACTTTATACTTTATGGATCTTGGTTCAGCTGCATGAAGCAGTTCCTGGGAAGAGATACAATAGATACGTGGAACTAGCGCAAGCTGCATTTGGTGAAAGACTGGGTGTTTGGCTTGCCCTCTTCCCTACTGTTTACCTATCTGCAGGGACTGCGACAGCTTTGATTCTCGTGGGAGGTGAAACCATGAAGTTGTTCTTTCAAATTGTTTGCGGTCCACTCTGTTCATCAAATCCTTTAACAACTGTAGAGTGGTATCTGGTTTTCACTTCCCTCTGCATTGTTCTATCCCAACTCCCGAACTTGAACTCCATTGCTGGACTCTCCCTCGTTGGAGCAGTGACAGCCATCACATACGCCACTATGGCATGGGTCCTCTCTGTAAGCCAACCAAGACCACCTTCGATTTCATATGAACCCATTTCATTGCCTTCCTACACAGCTTCTCTCTTTTCTGTCCTGAACGCCATGGGTATCATAGCATTTACTTTTAGAGGGCACAATTTAGTTCTAGAAATTCAGGCAACAATGCCATCAACCTTCAAGCACCCTGCTCATGTGCCAATGTGGAAAGGAGCAAAAGTTGCATATTTCTTCATAGCCATGTGCCTGTTCCCTATTGCAATTGGAGGTTTCTGGGCTTATGGAAACCTTATGCCATCGGGAGGAATGCTAAGTGCTCTATACGCCTTTCACATTCATGATATTCCAAGAGGACTTCTCGCCATGACGTTTCTCTTAGTCGTGTTCAACTGTTTAAGTAGTTTCCAGATATACTCAATGCCAGCATTCGATAGTTTTGAAGCAGGCTACACCAGCCGTACCAACCGACCATGCTCAATCTGGGTCCGTTCTGGATTCAGAATATTTTTCGGATTCGTTTCGTTCTTTATTGGAGTGGCGCTTCCGTTCCTGTCAAGTCTTGCAGGGTTGTTAGGAGGACTTACACTTCCGGTAACATTTGCTTATCCTTGCTTCATGTGGGTTCTAATAAAGAAGCCTACAAAGTATAGCTTTAACTGGTATTTCAACTGGATCCTTGGATGGTTAGGAGTTGCTTTTAGCTTGGCTTTTTCCATTGGAGGTATTTGGAGTATGGTCAATAATGGACTTAAACTCAGGTTCTTTAAGCCCAGCTAA

*NtLHT20*

ATGGAGGAAAGACCTGAGACTGAACTCATTTCAATACCAGCAACGCCACGTGCATCAACGCCTGAGATTCTAACGCCGTCAGGTCAAAGATCACCTAGGGGAGGACATACATCAACTGGAGCTTCTAATAAAGATGCTAAATCATGGACACCAACTTCATTTATTTCGCCTAGATTCTTGAGCCCTATTGGTACTCCAATGAAAAGGGTGTTGGTTAATATGAAAGGCTATTTGGAAGAAGTTGGTCATTTGACTAAGCTTAATCCTCAAGATGCTTGGCTTCCTATTACTGAGTCTCGTAATGGGAATGCTCACTATGCTGCTTTTCATAATCTCAATGCTGGTATTGGGTTTCAAGCTTTGGTCTTGCCTGTTGCTTTCTCCTTTCTTGGATGGAGTTGGGGAATAATTTCCTTAACTATAGCTTATTTCTGGCAACTCTATACTTTATGGATCTTGGTTCAGCTGCATGAAGCAGTTCCTGGGAAGAGATACAACAGATACGTGGAACTAGCGCAAGCAGCATTTGGTGAAAGACTGGGTGTTTGGCTTGCTCTCTTCCCTACTGTTTACCTATCTGCAGGGACTGCGACAGCTTTGATTCTCGTAGGAGGTGAAACCATGAAGTTGTTCTTTCAAATTGTTTGCGGTCCGCTGTGTTCCTCAAATCCTTTAACAACTGTGGAGTGGTATTTGGTTTTCACTTCCCTCTGCATTGTTCTATCCCAACTCCCAAACCTGAACTCCATTGCTGGACTCTCCCTCGTTGGAGCAGTGACAGCCATCACATACGCCACTATGGCATGGGTCCTCTCTGTAAGCCAACCAAGACCACCTTCGATTTCATATGAACCCATTTCATTGCCTTCCTACACAGCTTCTCTCTTTTCTGTCCTGAACGCCATGGGTATTATAGCATTTACTTTTAGAGGGCACAATTTAGTTCTTGAAATTCAGGCAACAATGCCGTCAACTTTCAAGCACCCAGCTCATGTGCCAATGTGGAAGGGAGCAAAAGTTGCGTATTTCTTCATAGCCATGTGCCTGTTCCCTATTGCCATTGGAGGCTTCTGGGCTTATGGAAACCTTATGCCATCGGGAGGAATGTTAAGTGCTCTATACGCCTTTCACATTCATGATATTCCGAGAGGACTTCTTGCCATGACATTTCTCTTAGTCGTATTCAACTGTTTAAGTAGCTTCCAGATATACTCAATGCCAGCATTCGACAGTTTTGAAGCAGGCTACACCAGCCGTACCAACCGACCATGCTCAATATGGGTCCGTTCCGGTTTCAGAATATTTTTTGGATTCGTTTCGTTCTTTATTGGAGTGGCGCTTCCGTTCCTGTCAAGTCTTGCAGGGTTGTTAGGAGGGCTTACACTTCCGGTAACATTTGCTTATCCTTGCTTCATGTGGGTTCTAATAAAGAAGCCTACAAAGTACAGCTTTAACTGGTATTTCAACTGGATCCTTGGATGGTTAGGAGTTGCTTTTAGCTTGGCTTTTTCTATTGGAGGAATTTGGAGTATGGTCAATAATGGACTTAAACTCAGGTTCTTTAAGCCCAGCTAA

*NtLHT21*

ATGACTGAAGTGGAGAAGAAAAAGAGAGGATCAGGATCTGAGGGAAGCAGTAATAAGGATCTGAACGATTGGCTACCAATCACATCATCAAGGAATGCAAAGTGGTACTATTCGGCGTTTCACAATGTCACTGCCATGGTTGGTGCTGGTGTTCTTGGCCTTCCTTATGCCATGTCTCAGTTGGGTTGGGGTGCTGGAGCAACAGTAATGGTATTATCATGGGTGATAACATTATACACCTTATGGCAAATGGTAGAGATGCATGAGATGGTTCCAGGTAAAAGATTTGACAGATACCATGAGTTGGGACAACATGCTTTTGGTGAAAAACTTGGGCTTTGGATTGTGGTTCCTCAGCAGCTAATGGTTGAAGTTGGAGTGAACATAGTGTATATGGTTACTGGTGGCAAATCCATTAAAAAGATATATGACACAGCTTGTCCTAGTTGTAGACCATTGAAAACCACCTATTTTATCATGATGTTTAGTTCTATTCACTTCTTCCTCTCCTATTGTCCCAATTTCAACTCCATTACTCTTGTCTCCTTCCTTGCTGCCATCATGTCTCTCAGTTATTCAACGATAGGTTGGGGAGCATCAGTACGTAAAGGAATAGCACCAGAGGTTGATTACAGTCCAAGGGCTTCAACAACAGCAGGAAGATTGTTTGGTTTCTTAAGTGCTTTAGGAGATGTTGCTTTTGCATTTGCTGGTCATAATGTTGTCTTGGAAATTCAGGCAACTATGCCTTCTTCTCCTGAGAAACCAGCCAAGAAACCTATGTGGAAAGGAGTCATTTTTGCCTATATCGTTGTGGCTTTGTGTTACTTTCCTGTGGCTTTTGTTGGCTATGCAGTTTTTGGGAAAAGTGTTGAGGATAATATCTTGATCTCCCTTGAGAAACCTGCTTGGCTTATTATCATTGCTAACGCCTTCGTTGTTGTCCATGTTATTGGAAGCTATCAGGTGTTTGCAATGGGTGTGTTTGACATGGTGGAATCTTACTTGGTGAAGCAAAGGAAATTCAGTCCAACTAAAACGCTACGGTTCATTGTTCGGACTAGTTATGTTGCCCTAACAATGGTTCTTGGTATAACATTCCCATTCTTTGGTGGGCTACTGGGTTTCTTCGGAGGATTTGCATTTGCTCCTACTACTTACTTTCTTCCTTGTATCATGTGGCTTGCAATCTACAAACCTAAAAAATTTGGCTTGTCCTGGTTCACTAATTGGATATGCATCATACTTGGTGTTCTTCTGATGATTTTAGCTCCCATTGGTGCCTTGAGGCAGATCATATTGCAAGCCAAGGATTACAAGTTCTATTCTTGA

*NtLHT22*

ATGCAACCTGCAACTATGGGAACGCAAACTCCAAATATTTCCAATTACTACTGCTCCAAGAGTGCTGATGAAAGATCTGCAGAAGAGAGGGCAATAGACGCATGGCTTCCTGTTACTTCAAACAGGAATGCGAAATGGTGGTATTCAGCTTTCCACAATGTTACTGCCATGGTCGGAGCTGGTGTCCTTGGTCTTCCTTATGCCATGGCACAACTTGGATGGGGACCAGGAGTAGCAGTGCTGGTGATTTCTTGGATTATAACATTTTACACATTATGGCAAATGGTTGAGATGCACGAAATGGTTCCTGGGAAACGTTTTGACAGATATCATGAACTTGGGCAGCATGCTTTTGGGAAAAAACTTGGGCTATGGATTATTGTGCCACAACAGTTGATTGTTGAAGTTGGAGTTGACATAGTTTATATGGTAACCGGAGGACAATCACTCCAGAAATTCTATGATCTAGTCTGTAAAAAAGATTGTAAAGACATAAAACTTACCTACTTCATTATGATCTTTGCCTCTGTCCATTTTGTTATCTCTCATCTTCCTGATTTCAATTCCATAGTAGGTGTGTCTTTGGCTGCAGCTGTCATGTCCTTAAGTTACTCAACAATTGCTTGGGGAGCTTCAGTTAAGAAAGGTGTAGTACCAGATGTGGAATATGGATACAAGGCAAAGTCAACAGCAGGAACAATTTTCAACTTTTTCAGTGCATTGGGAGATGTTGCTTTTGCTTATGCTGGCCATAATGTGGTGTTAGAAATTCAAGCTACAATCCCTTCAACACCTGAAAAGCCTTCAAAAGGACCTATGTGGAAAGGAGTTATTGTTGCTTATATAATTGTTGCTTTCTGTTATTTCCCTGTTGCTCTTATTGGCTACTGGATGTTTGGGAATCAAGTGAAAGACAACATTCTTAAGACTTTGGAGAAACCTACCTGGCTCATTGCTATGGCTAACTTGTTTGTTGTTATTCATGTTATTGGGAGTTATCAGATATATGCAATGCCAGTATTTGACATGATAGAAACAGTGCTTGTCAGAAAACTTAAGTTCAAGCCAAGCTGGATGTTGCGCTTTGTTACTAGGAACATTTATGTAGCTTTCACAATGTTTGTTGGCATTACCTTCCCTTTCTTCAATGGGCTGCTTGGATTCTTTGGAGGATTTGCTTTTGCCCCAACAACCTATTTTCTCCCTTGCATCATGTGGCTAGCAATCTGCAAACCAAAGAAATTCAGTCTCTCTTGGATTATTAATTGGATTTGCATTATTCTTGGAGTACTATTAATGGTTATAGCACCAATTGGTGGCCTAAGATCCATAATCATGCAAGCCAAGGGCTACAAATTTTACTCTTAA

*NtLHT23*

ATGGTTGAGAGTGGGAATAACTCCAATGATGGAAGGACAAATGAAGAAATCAATGATCTGAATAAGTGGCTGCCTATAACAGCTTCCCGAAAAGCCAAATGGTGGTATTCAGCATTCCATAATGTAACAGCCATTGTAGGAGCTGGTGTTCTAGGTTTACCATGTGCTGTGTCACAACTCGGCTGGATTCCAGGAATCGGGATGATAATAATTTCATGGAGTGTGACATTATACTCATTTTGGCAATTGGTTAATTTGCACGAGCATGTTCCGGGGAAGAGATTTGACAGATATCCTGAGTTAGGCAAACATGTATTTGGACTGAAGAGGGGTTACTGGATGGTGATGCCTCAACAGATAATTGTTCAAGTTGCCTGCGACATAGTATATATGGTTACAGGGGGGAAGTCTCTAAGGGAAAGTGTGAAGATGATGTTCCATTGGGGTCGCAAAATTAATCAAACTTACTATATTATGTTTTTTGGAGTTCTTCAATTGATACTATCCCAAGCTCCTAATTTTAATTCCTTGAAGGTGGTCTCTTTTACAGCAGCTGTTATGTCTTTGAGTTACTCAACAATTGCGTCAGTTGCGTCAATCATCAAAGGAATTGAGCATCCGAAAGCAGTTAATTATGGTCTTCGATCTCATACTACAGCTGGAATAATATTTGATATTTTCAACAGCTTAGGAACCATTGCATTTGCATTTGCTGGACATAGTGTTGCATTAGAAATTCAAGCAACCATCCCTTCAACCCCAGAAAGACCCTCTAAAAGTCCAATGTGGCGAGGTGTTGTCGTGGCTTATGCAATTGTTGCATTTTGCTATTTATCTGTTGCAGCCTCTGGATTCTGGGCTTTTGGCAATCTTGTGGCTGATGACGTCCTTGTTACATTAGAACACCCAAATTGGCTAATCTCTCTGGCAAACTTCATGGTGTTTTTGCATGTTCTTGGATCCTATCAGGTTTTTGCAATGCCTGTTTTTGACACGATTGAATCTTTCTTGGTTAAAAAGCGTCATTTCACTCCTGGGCGGCCTCTTCGCCTTATTGCCCGAAGCATTTATGTTGCTCCCATGCATGATGTGGCTTGTCAGCCAGAAACCTAA
