# Supplementary material for: Comprehensive analysis of the LHT gene family in tobacco and functional characterization of NtLHT22 involvement in amino acids homeostasis
Source: Front Plant Sci. 2022 Sep 13;13:927844. doi: 10.3389/fpls.2022.927844 (PMC9513474; doi:10.3389/fpls.2022.927844)
Supplement: Supplementary Table 3 — Primers used in this study. [file Table_3.docx]

**Table S3.** Primers used in this study.

| List of qRT-PCR primers used in this study | |
| --- | --- |
| primer name | sequence(5'-3') |
| NtLHT1-F | AGCCCTCCAAAGTTCCAATG |
| NtLHT1-R | TGCCCAATACCCGATGAAAG |
| NtLHT2-F | GGAATCAGCGTACACAACTAGG |
| NtLHT2-R | CTCCAATAAGCCCAGCAAAAC |
| NtLHT3-F | AGTTTCAAGGGTTGAGAGTGG |
| NtLHT3-R | TTTCGGGAAGCTGTTATAGGC |
| NtLHT4-F | ACGCTACGGTTTATTGTTCGG |
| NtLHT4-R | GCAAGCCACATGATACAAGG |
| NtLHT5-F | AGCAACCATCCCTTCAACC |
| NtLHT5-R | CAAAAGCCCAGAATCCAGAAG |
| NtLHT6-F | AAGCTACGATCCCTTCAACG |
| NtLHT6-R | GCACAGAATTCCCAAACACC |
| NtLHT7-F | CTGAGTGGATTGGGAGAAGTG |
| NtLHT7-R | CAGGTGTTGAAGGGATCGTAG |
| NtLHT8-F | AAGAACCCATCTCATGTGCC |
| NtLHT8-R | TCCAATGCACTCAATATCCCTC |
| NtLHT9-F | CTTGTTCTCCCCTTTGCTTTC |
| NtLHT9-R | GAGTTCCAGAGTTTGATTCGTG |
| NtLHT10-F | GTGTCCTCGGTCTTCCTTATG |
| NtLHT10-R | CAACCATTTGCCATAGCGTG |
| NtLHT11-F | ACCATTCCTTCAACACCCG |
| NtLHT11-R | AAAGCGACAGGGAGATAACAG |
| NtLHT12-F | GCCTATTTTGTCAATGCCCTG |
| NtLHT12-R | TTTCAAGTCCCACGAGCAC |
| NtLHT13-F | TCCAGATATACTCAATGCCAGC |
| NtLHT13-R | GCCACTCCAATAAAGAACGAAAC |
| NtLHT14-F | ACGCTACGGTTTATTGTTCGG |
| NtLHT14-R | GCAAGCCACATGATACAAGG |
| NtLHT15-F | AGAACCCATCTCATTTGCCC |
| NtLHT15-R | TCCAATGCACTCAATATCCCTC |
| NtLHT16-F | TCCAGATATACTCAATGCCAGC |
| NtLHT16-R | GCCACTCCAATAAAGAACGAAAC |
| NtLHT17-F | CTGAGTGGATTGGGAGAAGTG |
| NtLHT17-R | GGTGTTGAAGGGATTGTTGC |
| NtLHT18-F | TCCAGATATACTCAATGCCAGC |
| NtLHT18-R | GCCACTCCAATAAAGAACGAAAC |
| NtLHT19-F | TCTCTTTTCTGTCCTGAACGC |
| NtLHT19-R | CTTTTGCTCCCTTCCACATTG |
| NtLHT20-F | CCTCGGTCTTCCTTATGCTATG |
| NtLHT20-R | CTCAACCATTTGCCATAGTGTG |
| NtLHT21-F | ACGGTTCATTGTTCGGACTAG |
| NtLHT21-R | GCAAGCCACATGATACAAGG |
| NtLHT22-F | GCTTTCCACAATGTTACTGCC |
| NtLHT22-R | GTGCATCTCAACCATTTGCC |
| NtLHT23-F | ATCCCTTCAACCCCAGAAAG |
| NtLHT23-R | CAAGATTGCCAAAAGCCCAG |
| Primers used for NtLHT22-OE vector construct | |
| NtLHT22-OE-F | CAGGCTCCGCGGCCGCCACCATGCAACCTGCAAC  TATGGGAACGC |
| NtLHT22-OE-R | AAAGCTGGGTCGGCGCGCCCAGAGTAAAATTTGT  AGCCCTTGG |
