# Supplementary material for: Comprehensive analysis of the LHT gene family in tobacco and functional characterization of NtLHT22 involvement in amino acids homeostasis
Source: Front Plant Sci. 2022 Sep 13;13:927844. doi: 10.3389/fpls.2022.927844 (PMC9513474; doi:10.3389/fpls.2022.927844)
Supplement: Supplementary Table 4 — Ka and Ks ratios for seven paralogous NtLHT genes. [file Table_4.docx]

| Gene 1 | Gene 2 | Ka | Ks | Ka/Ks | Gene Duplications |
| --- | --- | --- | --- | --- | --- |
| *NtLHT1* | *NtLHT12* | 0.027 | 0.048 | 0.562 | Segmental |
| *NtLHT5* | *NtLHT23* | 0.013 | 0.045 | 0.289 | Segmental |
| *NtLHT6* | *NtLHT10* | 0.013 | 0.021 | 0.619 | Segmental |
| *NtLHT6* | *NtLHT22* | 0.026 | 0.059 | 0.441 | Segmental |
| *NtLHT8* | *NtLHT15* | 0.039 | 0.048 | 0.812 | Segmental |
| *NtLHT14* | *NtLHT21* | 0.036 | 0.045 | 0.800 | Segmental |
| *NtLHT6* | *NtLHT7* | 0.053 | 0.059 | 0.898 | Tandem |

Table S4. Ka and Ks ratios for seven paralogous *NtLHT* genes
