# Supplementary material for: Comprehensive analysis of the LHT gene family in tobacco and functional characterization of NtLHT22 involvement in amino acids homeostasis
Source: Front Plant Sci. 2022 Sep 13;13:927844. doi: 10.3389/fpls.2022.927844 (PMC9513474; doi:10.3389/fpls.2022.927844)
Supplement: Supplementary Table 5 — Collinear LHT genes between tobacco, tea, Arabidopsis, and rice. [file Table_5.docx]

Table S5 collinear *LHT* genes among tobacco, rice, tea and *Arabidopsis*

| tobacco and rice | |
| --- | --- |
| *NtLHT14* | *OsLHT2* |
| *NtLHT21* | *OsLHT2* |

| tobacco and *Arabidopsis* | |
| --- | --- |
| *NtLHT1* | *AtLHT9* |
| *NtLHT2* | *AtLHT7* |
| *NtLHT12* | *AtLHT9* |

| tobacco and tea | |
| --- | --- |
| *NtLHT8* | *CsLHT3* |
| *NtLHT8* | *CsLHT5* |
| *NtLHT19* | *CsLHT4* |
| *NtLHT19* | *CsLHT7* |
